# DNA hypomethylation and aberrant expression of the human endogenous retrovirus ERVWE1/syncytin-1 in seminomas

**DOI:** 10.1186/s12977-017-0342-9

**Published:** 2017-03-17

**Authors:** Martina Benešová, Kateřina Trejbalová, Denisa Kovářová, Zdenka Vernerová, Tomáš Hron, Dana Kučerová, Jiří Hejnar

**Affiliations:** 1 Institute of Molecular Genetics, Czech Academy of Sciences, Videnska 1083, 14220 Prague 4, Czech Republic; 2 Department of Pathology, Third Faculty of Medicine, Charles University, Prague, Czech Republic

**Keywords:** Human endogenous retrovirus, ERVWE1, Germ cell tumor, Seminoma, Promoter DNA methylation, 5-Hydroxymethylcytosine, Transcription, RNA splicing

## Abstract

**Background:**

Syncytin-1 and 2, human fusogenic glycoproteins encoded by the env genes of the endogenous retroviral loci ERVWE1 and ERVFRDE1, respectively, contribute to the differentiation of multinucleated syncytiotrophoblast in chorionic villi. In non-trophoblastic cells, however, the expression of syncytins has to be suppressed to avoid potential pathogenic effects. Previously, we have shown that the transcriptional suppression of ERVWE1 promoter is controlled epigenetically by DNA methylation and chromatin modifications. In this study, we describe the aberrant expression of syncytin-1 in biopsies of testicular germ cell tumors.

**Results:**

We found efficient expression and splicing of syncytin-1 in seminomas and mixed germ cell tumors with seminoma component. Although another fusogenic gene, syncytin-2 was also derepressed in seminomas, its expression was significantly lower than that of syncytin-1. Neither the transcription factor GCM1 nor the increased copy number of ERVWE1 were sufficient for this aberrant expression of syncytin-1 in seminomas. In accordance with our recent finding of the highly increased expression of TET1 dioxygenase in most seminomas, the ERVWE1 promoter was significantly hypomethylated in comparison with the matched controls. In contrast, 5-hydroxymethylcytosine levels were not detectable at the ERVWE1 promoter. We further describe that another endogenous retroviral element adjacent to ERVWE1 remains transcriptionally suppressed and two additional HERV-W family members are only slightly upregulated in seminomas.

**Conclusions:**

We conclude that DNA demethylation of the ERVWE1 promoter in seminomas is a prerequisite for syncytin-1 derepression. We propose the spliced syncytin-1 expression as a marker of seminoma and suggest that aberrant expression of endogenous retroviruses might be a correlate of the hypomethylated genome of seminomas.

**Electronic supplementary material:**

The online version of this article (doi:10.1186/s12977-017-0342-9) contains supplementary material, which is available to authorized users.

## Background

Human endogenous retroviruses (HERVs) represent the remnants of the past retroviral infections in the human lineage that penetrated the germ line leaving behind the heritable proviral copies in the human genome. With a few exceptions, HERVs have become defective by gradual accumulation of inactivating mutations in their open reading frames. Transcription of most HERV copies is silenced by epigenetic mechanisms such as DNA methylation and association with histone molecules marked by suppressive modifications.

Although endogenous retroviruses are widely employed in genome evolution as a source of regulatory sequences and interfere with new exogenous infections, there are few documented captures of intact retrovirus-encoded proteins for cellular functions. Syncytin-1 and -2 are retroviral envelope glycoproteins whose original receptor-mediated virus-to-cell fusion capacity evolved towards cell-to-cell fusion [1–3]. This fusogenic potential unfolds in all respects in placenta where syncytins are indispensable for syncytiotrophoblast differentiation. Only recently, the contribution of fusogenic syncytins to myogenesis and osteoclast fusion was suggested for mouse and human [1, 4]. On the other hand, the expression of syncytins must be suppressed in non-syncytial tissues, and, therefore, the capture of fusogenic retroviral envelope glycoproteins has to be accompanied by the gain of tissue-specific regulation. The importance of tissue-specific regulation of syncytin-1 is even underscored by the ubiquitous expression of its specific receptors, the human sodium-dependent neutral amino acid transporters type 1 and 2 (ASCT1, also known as SLC1A4, and ASCT2/SLC1A5) [5, 6].

Syncytin-1 is encoded by the *env* gene of the ERVWE1 provirus (NCBI accepted name ERVW1), a prototype member of the HERV-W family, localized on chromosome 7 [7, 8]. Both *cis* and *trans* regulatory circuits have been described [9, 10]; however, the tissue-specificity of syncytin-1 expression is controlled epigenetically. We and others demonstrated that the ERVWE1 transcription was regulated by DNA methylation and trimethylation of H3K9 of the ERVWE1 5′ LTR [11–14]. Furthermore, we showed that the splicing of ERVWE1 mRNA occurrs in trophoblastic but not in non-placental cells [13] and serves as an additional control mechanism. Syncytin-2 encoded by the *env* gene of a unique member of the HERV-FRD family, ERVFRDE1 (NCBI accepted name ERVFRD1), is also important for the fusion of human cytotrophoblast [15]. Like ERVWE1, ERVFRDE1 is also regulated epigenetically [14, 16], but the role of DNA methylation and splicing is less understood.

Aberrant expression of Syncytin-1 has been reported in multiple tumor types, but epigenetically-based evidence is available just for endometrial carcinomas [17, 18] and several samples of testicular tumors without exact characterization [13, 14]. Testicular germ cell tumors (GCT) originate from embryonic primordial germ cells (PGC) or gonocytes as in situ neoplasias and transform into seminomas. Non-seminomas require further development, probably with reprogramming and dedifferentiation steps [19]. GCTs maintain, to a various extent, the epigenetic characteristics of their PGC precursors, i.e., DNA hypomethylation and low levels of H3K9 trimethylation [20, 21]. Comparison of seminomas and non-seminomas showed a lower level of genome methylation in seminomas and increased methylation in non-seminomas [22–26]. This again suggests that seminomas and non-seminomas arise in distinct periods of the PGC development with different degrees of cell differentiation. Recently published analyses of the transcriptional and epigenetic landscape during human PGC development revealed progressive erasure of DNA methylation not only from the global genome, but also from the transposable elements [27–29].

The deeply hypomethylated genome of seminoma cells has been recently correlated with elevated expression of the TET1 enzyme in GCTs [30]. The DNA demethylation activity of TET dioxygenases proceeds through 5-hydroxymethylcytosine (5-hmC) intermediate [31], which subsequently converts to 5-formylcytosine (5-fC), 5-carboxycytosine and unmodified cytosine (C) [32, 33]. At least in mouse, Tet1 and Tet2 expression has been observed in late PGCs [34] and together with the repression of *de novo* DNA methyltransferases [35] create the hypomethylated germ line genome.

Based on the knowledge of DNA hypomethylation in GCTs and the observation of syncytin-1 expression in testicular tumors [13, 14], we explored the ERVWE1 expression systematically within a panel of GCTs with particular respect given to discrimination between seminomas and non-seminoma GCTs. In addition, we included several samples of lymphomas and endometrial carcinomas in our analysis because of (1) the presence of multinuclear giant Reed-Sternberg cells in Hodgkin lymphomas [36], (2) the detection of full-length ERVWE1 mRNA in endometrial carcinomas [17, 18, 37], and (3) the recently described increased expression of DNA demethylating dioxygenases TET2 and TET3 in seminomas, lymphomas and endometrial carcinomas [30] that could contribute to ERVWE1 transcriptional derepression. We estimated, for the first time, the levels of both 5-methylcytosine (5-mC) and 5-hmC modifications at the ERVWE1 promoter within the 5′LTR. We also examined the transcription level of four other endogenous retroviruses, including ERVFRDE1, in the GCTs.

## Methods

### Tissue samples

Testicular samples were collected from patients who were surgically treated at the Institute of Urology, University Hospital Kralovske Vinohrady, and Third Faculty of Medicine, Charles University between years 2011–2015. Histological classification and TNM staging were done in accordance with WHO classification [19]. Lymphoma samples were acquired from the Department of Otorhinolaryngology, University Hospital Kralovske Vinohrady, and Department of Surgery, University Hospital Kralovske Vinohrady. Endometrial carcinomas and six healthy term placental samples were obtained from the Department of Obstetrics and Gynecology, University Hospital Kralovske Vinohrady. Immediately after the surgery, the samples were frozen and stored in −80 °C.

Our extensive set of post-pubertal testicular malignant GCT samples contained 30 pure seminomas, one scar after the seminoma, and 17 non-seminomas. The non-seminomas included 12 mixed GCTs, two pure embryonal carcinomas, two pure teratomas, and one pure yolk sac tumor. For the majority of cancer biopsies, the tumor-matched controls were collected from the adjacent tissue not affected macroscopically with the GCT. In some cases, two biopsies from distinct parts of the tumor were analyzed. The set also contained ten samples of the testes not diagnosed with the GCTs (termed non-GCT controls throughout the text). The non-GCT controls included five samples of atrophic testes, one sample with testicular necrotic tissue, two testis after ischemia–reperfusion injury, and two testis without GCT. Our set of malignant human tumor samples further contained tumors of non-germ cell origin, specifically three Hodgkin lymphomas and five non-Hodgkin lymphomas, and seven endometrial carcinomas, including six endometrioid and one non-endometrioid. Description of tumor samples is indicated in Additional file 1: Table S1.

### Cell culture procedures

Choriocarcinoma BeWo cells were maintained in F-12 and MEM-D media mixed 1:1 (Sigma) supplemented with 1% NaHCO_3_, 10% FBS. Seminoma TCam-2 cells were maintained in RPMI-1640 (Sigma) supplemented with 10% FBS and 0.3 mg/ml l-glutamine. A mix of penicillin and streptomycin (0.1 mg/ml each) was added to both cell lines in culture. Cells were kept at 37 °C in humidified atmosphere of 5% CO_2_.

### Quantitative RT-PCR

**Fig. 1:**
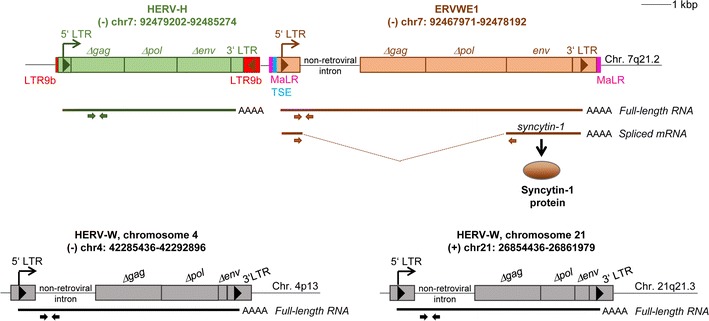
Schematic illustration of the examined human endogenous retroviruses, ERVWE1, the adjacent endogenous retroelement HERV-H at chromosome 7q21.2, and two HERV-W loci on chromosomes 4 and 21. Human endogenous retrovirus ERVWE1 includes 5′LTR containing the enhancer/promoter sequence, 3′LTR, an intron of cellular origin located downstream of 5′LTR, the *Δgag*-*Δpol* gene and the *env* gene with an intact open reading frame. Two RNA variants of ERVWE1 can be produced (depicted by bold lines): full-length RNA, which includes all three genes mentioned above and monocistronic spliced mRNA containing the *env* gene. The Syncytin-1 protein can be produced only from the spliced form. ERVWE1 itself integrated into MaLR LTR whose parts are located on both sides of the ERVWE1, Trophoblast-specific enhancer (TSE) located upstream of the ERVWE1 5′ LTR contains a GCM1 binding site and is required for placenta-specific expression of ERVWE1. Another retroelement in the close proximity of ERVWE1, the human endogenous retrovirus HERV-H, consists of 5′ and 3′ LTR and the *gag*-*pol* gene, and is integrated within the LTR9b retroelement. In addition, we examined expression of two HERV-W elements on chromosomes 4 and 21. Both contain 5′ and 3′ LTRs, an intron of cellular origin located downstream of 5′LTR, the ∆*gag*-∆*pol* gene and the ∆*env* gene. Pairs of primes used for the qRT-PCR analysis are depicted by arrows. Length-scale bar indicates 1 kbp. Chromosomal positions assigned according to the genome assembly GRCh38/gh38, December 2013

Total RNA from tissue samples was isolated using the RNAzol^®^ RT reagent (Molecular Research Centre, INC.) according to manufacturer’s instructions. One µg of total RNA was reverse-transcribed by random primers (Promega) and Protoscript II Reverse Transcriptase (New England Biolabs) following the manufacturer’s protocol. RT-minus reaction that served as a control of residual DNA contamination in the RNA sample did not contain the Reverse Transcriptase. Both absolute and relative qRT-PCR analyses of the synthetized cDNA were employed to quantify the mRNA expression of the genes of interest (ERVWE1, ERVFRDE1, GCM1, ASCT1, ASCT2, HERV-Ws on chromosomes 4 and 21) and RNA polymerase II, subunit A (POLR2A), which served for normalization as a housekeeping control. MESA GREEN qRT-PCR Master Mix Plus for SYBR Assay (Eurogentec) with a CFX1000 cycler and CFX Manager Software 3.1 (both BioRad) were applied. All reactions were run in triplicate and the average Cts were used for quantitation. The negative control contained water instead of cDNA. Calibration curves derived from serial ten-fold dilutions of the known quantity of ERVWE1, syncytin-1, ERVFRDE1, syncytin-2, GCM1, HERV-Ws on chromosomes 4 and 21, and POLR2A molecules were used to calculate the absolute numbers of mRNA molecules. The data were normalized as the percentage of mRNA of the POLR2A in the sample. Full-length and spliced forms of ERVWE1 and ERVFRDE1 were discriminated by using the same forward primers localized in 5′LTR in combination with the reverse primers localized either in the 5′UTR region or in the coding sequence of *env* (Fig. 1). The specificity of all primers was thoroughly verified by sequencing multiple random clones of the respective PCR products. To correctly quantify the amount of the full-length ERVWE1, ERVFRDE1 and HERV-Ws on chromosome 4 and 21 RNAs that do not contain introns between the pair of primers, the data obtained from RT-minus controls were subtracted from the total amount measured in the RT-plus sample. In all experiments, the RT-minus controls reached less than 5% of RT-plus samples. Relative quantification was used for the analysis of ASCT1 and ASCT2 expression and was also normalized to the corresponding POLR2A. For all qRT-PCR analyses, the common protocol was applied: 40 cycles of 95 °C 15 s, primer annealing 20 s, 72 °C 30 s, and fluorescence reading. The sequences of primers and annealing temperatures are indicated in Additional file 2: Table S2.

In order to confirm the results normalized to POLR2A, we additionally normalized expressions of the full-length ERVWE1, spliced syncytin-1, full-length ERVFRDE1 and spliced syncytin-2 in all samples also to the TATA-box binding protein (TBP) gene. The calibration curve derived from serial ten-fold dilutions of the known quantity of TBP molecules was used to calculate the absolute numbers of mRNA molecules. The results were proportional to the POLR2A normalization (see Additional file 3: Fig. S1 (A) to (D)) corresponding to the primer efficiency of 93 and 95% for POLR2A and TBP, respectively.

### Droplet digital PCR

To analyze the copy number variation and expression of different human endogenous retroviruses, we performed absolute quantification by means of droplet digital PCR (ddPCR). For the copy number variation we used total chromosomal DNA, for the expression analysis we used the synthetized cDNA. The PCR reaction contained ddPCR™ Supermix for Probes and Droplet Generation Oil for Probes and was performed by means of QX100™ Droplet Digital™ PCR System (all BioRad). The protocol for the copy number analysis consisted of: 95 °C 10 min, 95 °C 30 s–60 °C 60 s—40 cycles. For the qRT-PCR analysis, ddPCR protocol was following: 95 °C 10 min, 95 °C 30 s–57 °C 30 s–72 °C 30 s—43 cycles. The data were analyzed with QuantaSoft™ software. All reactions were run in duplicate and the average concentrations were used for quantification. The negative control contained water instead of DNA or cDNA. For both analyses, the same PCR reaction contained two pairs of primers and two probes in parallel to quantify the gene of interest and the gene used for normalization at the same time. The copy number variation of ERVWE1 was normalized according to the RPP30 (Ribonuclease P/MRP Subunit P30) copy number. The absolute expression of human endogenous retrovirus HERV-H was normalized according to the POLR2A expression, and respective RT-minus controls reaching up to 5% of their RT-plus counterparts were subtracted. The specificity of primers and probes was verified by sequencing the amplified products. Sequences of primers and probes used for ddPCR analysis are indicated in Additional file 2: Table S2.

### Chromosomal DNA isolation

Samples of total genomic DNA from the tissues, TCam-2, and BeWo cell lines were isolated using Proteinase K, RNase A, phenol–chloroform extraction and ethanol precipitation.

### Bisulfite and oxidative bisulfite sequencing

Simple bisulfite treatment does not distinguish between 5-mC and 5-hmC, and keeps both 5-mCs and 5-hmCs as Cs while converting all unmodified Cs into uracils (U). On the other hand, oxidative DNA treatment ensures quantitative conversion of 5-hmC bases into 5-fCs. The bisulfite treatment of oxidized DNA converts the 5-fCs and the non-modified Cs into Us, while only the 5-mCs are kept as non-converted Cs. Therefore, to identify the percentage of 5-hmC in a specific sequence, the percentage of 5-mC obtained after oxidative treatment plus bisulfite sequencing must be subtracted from the percentage of 5-modified C obtained after the simple bisulfite treatment. One µg of genomic DNA was used for simple bisulfite conversion or oxidative treatment plus bisulfite conversion by means of TrueMethyl^®^ Seq kit (CEGX, Cambridge Epigenetix) applied according to the protocol recommended by the manufacturer. Bisulfite-treated DNA was amplified by PCR specific for 5′ LTR in a 50-µl reaction mixture using ERVWE1-BIS-FW and ERVWE1-BIS-RV primers. See Additional file 2: Table S2 for the primer sequences. The bisulfite-specific sense primers contained thymine and the antisense primers adenine instead of C in positions complementary to non-methylable C (i.e., C out of CpG dinucleotides). One 50-μl reaction contained: 2 μl of bisulfite/oxidative bisulfite-treated product, 2.5 mM MgCl_2_, 2 μg HotStart-IT^®^ Binding Protein (Affymetrix), 0.32 μM primers, 2 U Platinum Taq polymerase (ThermoFisher Scientific) and 0.2 mM dNTPs (Promega). The following PCR program was applied: 95 °C 5 min, 95 °C 50 s–58 °C 2 min–68 °C 1 min 30 s—25 cycles; 95 °C 45 s–54 °C 2 min–68 °C 1 min 30 s—15 cycles and 68 °C 10 min. At least three primary PCRs were performed for each sample to exclude amplification of one template molecule. Non-converted DNA did not provide bands. Several non-template controls were included in each bisulfite PCR reaction. Amplification products were cloned in the pGEM-T-EasyVector System (Promega) and sequenced. Analysis was performed using the Quma (Quantification tool for Methylation Analysis) software (http://quma.cdb.riken.jp/). Only PCR clones with at least 95% conversion of cytosines outside CpGs were taken into account. When more converted molecules with identical sequences were obtained, only one was used for calculation of the methylated CpG percentage to minimize the bias originating from the preferential amplification of one molecule.

The efficiency of 5-hmC to 5-fC oxidation was controlled using the 5-hmC-containing synthetic probe. The corresponding region of the ERVWE1 promoter was amplified by PCR from genomic DNA using the dNTP mix with dhmCTP replacing the dCTP (see Additional file 2: Table S2 for the primer sequences ERVWE1-nonBIS-FW and ERVWE1-nonBIS-RV). The amplified product of 538 bp containing 155 5-hmCs was isolated from the agarose gel and 100 ng of the 5-hmC-containing fragment was subjected to oxidative bisulfite treatment by means of TrueMethyl^®^ Seq kit (CEGX, Cambridge Epigenetix). Thereafter, PCR was performed using the bisulfite-specific ERVWE1 primers (see Additional file 2: Table S2, primers ERVWE1-BIS-FW and RV). Amplification products of three primary PCRs were cloned in the pGEM-T-Easy vector and sequenced. Analysis was performed using the Quma software and all PCR clones were taken into account regardless of the C conversion rate. Overall 5-hmC to U conversion levels of 97.9 to 99.3% were observed in individual molecules of the oxidation controls indicating efficient oxidation.

### Statistical analysis

For statistical analysis of data GraphPad Prism software, version 5.04, was used and two-tailed Mann–Whitney test was applied. Significance was assigned as follows: **** for P-values <0.0001, *** for P-values <0.001, ** for P-values <0.01, * for P-values <0.05.

## Results

### Derepression of ERVWE1 transcription in seminoma and other GCTs

In order to assess the ERVWE1 expression in the GCTs with particular respect to seminomas, we employed qRT-PCR and quantified the absolute levels of full-length ERVWE1 RNA (Fig. 1) in our panel of tumor samples and in tumor-matched controls (Additional file 1: Table S1). In total, 30 pure seminomas, one scar after the seminoma, and 17 non-seminoma GCTs, mostly mixed GCTs, were examined.

**Fig. 2:**
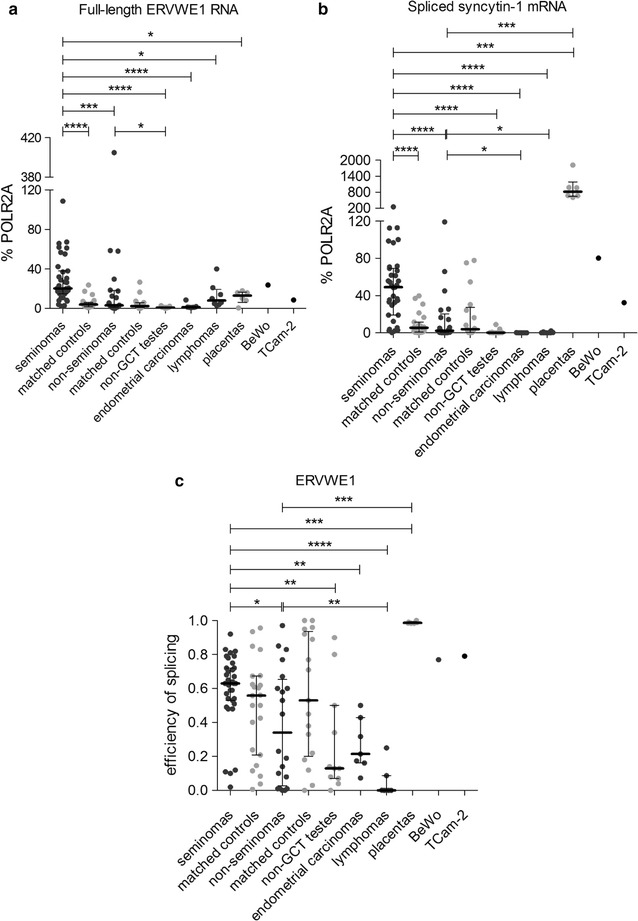
Expression analysis of the ERVWE1 locus. Expression from the ERVWE1 locus was analyzed by qRT-PCR in the panel of tumor samples. Both the full-length RNA (**a**) and spliced syncytin-1 mRNA (**b**) forms were quantified. All the data were normalized to % POLR2A. Each sample is represented by a dot and was measured as a technical triplicate. In each column, median with interquartile range is depicted. Splicing efficiency is shown in (**c**). Each sample is depicted by a dot which represents the ratio of spliced syncytin-1 mRNA to the sum of full-length RNA and spliced mRNA in the analyzed sample. Significance was assigned as follows: **** for P-values <0.0001, *** for P-values <0.0001, ** for P-values <0.01, * for P-values <0.05

We observed significantly increased levels of full-length ERVWE1 RNA in seminomas with median 20% of POLR2A in comparison with seminoma-matched controls (4% of POLR2A) and non-seminoma GCTs (3% of POLR2A, Fig. 2a). Median of the ERVWE1 expression in seminomas was higher than in the seminoma cell line TCam-2 (9% of POLR2A) and was comparable with BeWo cells (24% of POLR2A), the fusogenic cell line of choriocarcinoma origin (Fig. 2a). ERVWE1 full-length RNA levels in the non-seminomas were comparable with non-seminoma-matched controls (2% of POLR2A), but higher than in the non-GCT testes (1% of POLR2A, Fig. 2a). Interestingly, among the non-seminoma GCTs, the maximum amount of full-length ERVWE1 RNA (404% of POLR2A) was detected in the mixed GCT T43 consisting of 40% choriocarcinoma, 30% embryonal carcinoma and 30% yolk sac tumor components. Two other mixed GCTs with superior expression of ERVWE1 contained the seminoma components (T32 with 80% seminoma and 20% embryonal carcinoma component, and T33 with 60–70% seminoma, 25–30% teratoma, and 15% embryonal carcinoma component).

On the other hand, the analyzed samples of endometrial carcinomas and lymphomas displayed a significantly lower level of full-length ERVWE1 RNA (median 1 and 10% of POLR2A, respectively) than the seminomas (Fig. 2a). No significant differences in ERVWE1 RNA levels were observed between Hodgkin and non-Hodgkin lymphomas (median 14 and 5% of POLR2A, respectively, Additional file 4: Fig. S2). Unexpectedly, placenta samples also displayed a low level of full-length ERVWE1 RNA (median 13% of POLR2A, Fig. 2a).

In conclusion, seminomas displayed significantly higher levels of non-spliced ERVWE1 RNA in comparison with the seminoma-matched controls and also with the non-seminomas or endometrial carcinomas and lymphomas.

### Efficient splicing of ERVWE1 RNA in seminoma

Fusogenic Syncytin-1 can be translated only from spliced ERVWE1 env mRNA (Fig. 1). Therefore, we quantified the absolute levels of spliced syncytin-1 mRNA in GCTs employing the splice-specific qRT-PCR described previously [13].

We detected a very high level of syncytin-1 mRNA in seminomas (median 49% of POLR2A), BeWo cells (80% of POLR2A), and particularly placenta (median 820% of POLR2A, Fig. 2b). BeWo cells and placentas validated our splice-specific qRT-PCR assay because Syncytin-1 has been shown to be placenta-specific [3] and Syncytin-1-mediated cell-to-cell fusion was described in BeWo cells. Syncytin-1 mRNA expression in TCam-2 cell line reached 32% of POLR2A. The level of syncytin-1 mRNA in seminomas was significantly higher than in the seminoma-matched controls (5% of POLR2A) and non-seminoma GCTs (2% of POLR2A, Fig. 2b). Importantly, two mixed GCTs with the most abundant expression of full-length ERVWE1 RNA, T33 and T32, also displayed high levels of spliced syncytin-1 mRNA. Both tumors contained a large proportion of seminoma. In contrast, T43 with the highest level of full-length ERVWE1 RNA displayed only a low level of spliced syncytin-1 mRNA (3% of POLR2A). This mixed GCT lacked any seminoma component. Only negligible levels of the spliced syncytin-1 mRNA were found in the non-GCT testes, endometrial carcinomas and lymphomas (Fig. 2b). No significant differences in syncytin-1 mRNA levels were observed between Hodgkin and non-Hodgkin lymphomas (Additional file 4: Fig. S2).

We further determined the splicing efficiency as a ratio of spliced syncytin-1 mRNA to the sum of spliced and full-length ERVWE1 RNA in the same sample. Despite significantly higher amounts of ERVWE1 RNA and syncytin-1 mRNA in seminomas versus seminoma-matched controls, the efficiency of splicing in seminomas and seminoma-matched controls did not differ significantly (Fig. 2c). However, the efficiency of splicing in seminomas (median 0.63) was significantly higher in comparison with the non-seminomas (median 0.34, Fig. 2c). The 0.99, 0.79 and 0.77 splicing efficiencies were found in placentas, TCam-2 and BeWo cell lines, respectively. On the other hand, the lowest efficiency of splicing was detected in the non-GCT testes (median 0.13), endometrial carcinomas (median 0.21) and lymphomas (median 0.00).

Our results showed that, similarly to the non-spliced ERVWE1 RNA, the levels of the spliced syncytin-1 mRNA were upregulated in seminomas over the seminoma-matched controls and, again, over the non-seminomas. In seminomas, the ERVWE1 RNA was transcribed at high intensity, and the majority of this RNA was efficiently spliced into the syncytin-1 mRNA.

### Derepression of ERVFRDE1 expression in seminoma

ERVFRDE1 is the second endogenous retrovirus encoding placenta-specific, fusogenic glycoprotein Syncytin-2. Therefore, we examined the expression of both full-length and spliced ERVFRDE1 RNAs in our panel of seminomas and non-seminoma GCTs.

**Fig. 3:**
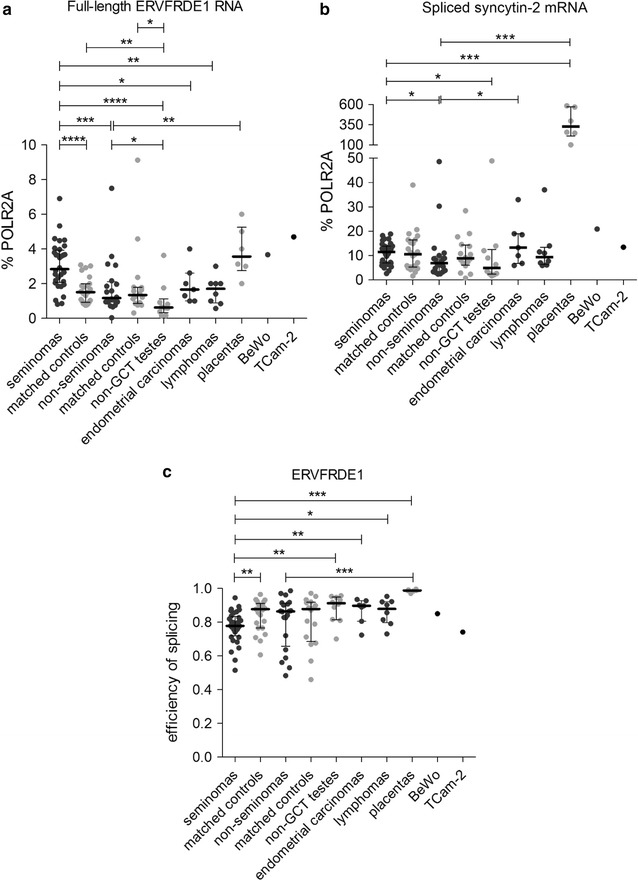
Expression analysis of the ERVFRDE1 locus. Expression from the ERVFRDE1 locus was analyzed by qRT-PCR in the *panel* of tumor samples. Both the full-length RNA (**a**) and spliced syncytin-2 mRNA (**b**) forms were quantified. All the data were normalized to % POLR2A. Each sample is represented by a *dot* and was measured as a technical triplicate. In each column, median with interquartile range is depicted. Splicing efficiency is shown in *panel* (**c**). Each sample is depicted by a dot which represents the ratio of spliced mRNA to the sum of full-length RNA and spliced mRNA in the analyzed sample. Significance was assigned as follows: **** for P-values <0.0001, *** for P-values <0.001, ** for P-values <0.01, * for P-values <0.05

The level of full-length ERVFRDE1 RNA in seminomas, placentas, TCam-2 and BeWo cells was low (median 3, 4, 5 and 4% of POLR2A, respectively), but significantly higher than in seminoma-matched controls and, importantly, non-seminoma GCTs (1.5 and 1.2% of POLR2A, respectively, Fig. 3a). Similarly to ERVWE1, the levels of spliced syncytin-2 mRNA in seminomas (median 12% of POLR2A) were higher than in non-seminoma GCTs (median 7% of POLR2A) and the non-GCT controls (median 5% of POLR2A, Fig. 3b). In contrast to ERVWE1, the levels of spliced syncytin-2 mRNA in seminomas were not elevated over the seminoma-matched controls (median 11% POLR2A, Fig. 3b). The levels of ERVFRDE1 RNA in Hodgkin and non-Hodgkin lymphomas were similar, but the levels of spliced syncytin-2 mRNA were higher in the non-Hodgkin lymphomas (median 12% of POLR2A) than in the Hodgkin lymphomas (median 6% of POLR2A, Additional file 4: Fig. S2). Placentas, that served as a positive control, showed the highest expression of spliced syncytin-2 mRNA (median 328% of POLR2A, Fig. 3b).

We further compared the efficiency of ERVFRDE1 splicing and found no significant differences between the seminomas and non-seminomas (Fig. 3c). Nevertheless, the efficiency of ERVFRDE1 splicing was lower in seminomas than in the seminoma-matched controls or the non-GCT testes, endometrial carcinomas or lymphomas (Fig. 3c). As in the case of ERVWE1, placentas displayed very high efficiency of ERVFRDE1 RNA splicing (median 0.99). Interestingly, the splicing efficiency of ERVFRDE1 RNA in both seminomas and non-seminomas was higher than that of ERVWE1 RNA (Additional file 5: Fig. S3).

Overall, elevated levels of the full-length ERVFRDE1 RNA and spliced syncytin-2 mRNA were found in seminomas in comparison to non-seminoma GCTs. When comparing the levels of the full-length and spliced forms of ERVWE1 versus ERVFRDE1 in the non-seminomas, the spliced syncytin-2 mRNA was slightly elevated over the spliced syncytin-1 mRNA (P > 0.05). On the other hand, in the seminomas, the levels of both non-spliced ERVWE1 RNA and spliced syncytin-1 mRNA were significantly higher than the levels of the non-spliced ERVFRDE1 RNA and spliced syncytin-2 mRNA, respectively (P > 0.0001 in both cases, Additional file 6: Fig. S4A and B). These results underscore the significance of ERVWE1 and syncytin-1 overexpression in seminomas.

### ASCT1 and ASCT2, the receptors of Syncytin-1, are not overexpressed in GCTs

**Fig. 4:**
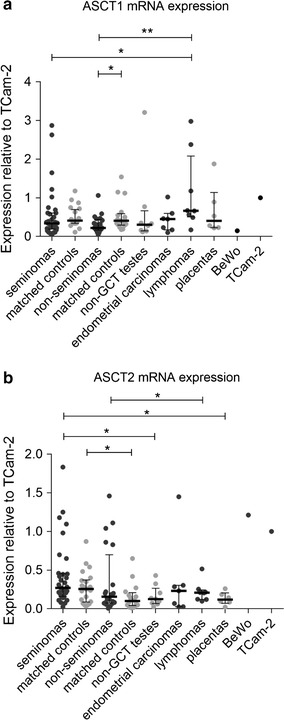
Human ASCT1 and ASCT2 mRNA expression analysis. The human ASCT1 (**a**) and ASCT2 (**b**) mRNA levels were measured by qRT-PCR. All the data were normalized to the expression in TCam-2 cells. Each sample is represented by a dot and was measured as a technical triplicate. In each column, median with interquartile range is depicted. Significance was assigned as follows: ** for P-values <0.01, * for P-values <0.05

The fusogenic capacity of Syncytin-1 requires interaction with its specific receptors ASCT1 and ASCT2. To assess the fusogenic potential of GCTs, we quantified the expression of ASCT1 and ASCT2 mRNA in our panel of seminomas and non-seminomas and compared it with the BeWo cell line and placenta samples, both characterized by efficient cell-to-cell fusion and Syncytin-1 expression. The expression of both receptors was quantified relatively to their expression in the TCam-2 cell line that displayed comparable and relatively high expression of both receptors (Fig. 4a, b). The relative levels of ASCT1 and ASCT2 mRNA in seminomas were low (median 0.34 and 0.27 of TCam-2 expression, respectively), and no significant difference from the seminoma-matched controls or non-seminomas was detected (Fig. 4a, b). Nevertheless, seminomas showed higher expression of ASCT2 than the non-GCT controls (median 0.13 of TCam-2 expression, Fig. 4b). Non-seminomas even displayed lower levels of ASCT1 mRNA than the matched controls (Fig. 4a). Placentas displayed ASCT1 mRNA levels comparable with seminomas (median 0.4 of TCam-2 expression, Fig. 4a) but the ASCT2 mRNA levels were lower than in the seminomas (median 0.1 of TCam-2 expression, Fig. 4b). ASCT1 mRNA expression in the BeWo cell line was lower than in placentas (0.2 of TCam-2 expression, Fig. 4a) but the ASCT2 expression was comparable with the TCam-2 cell line (1.2 of TCam-2 expression, Fig. 4b). Our results showed that seminomas do not express elevated levels of ASCT1 or ASCT2 mRNA over the matched controls or over the non-seminomas.

### Copy number of ERVWE1 in seminoma

Next, in order to determine the mechanism of ERVWE1 overexpression in seminomas, we quantified the ERVWE1 copy number using droplet-digital PCR. We examined the genomic DNA of five seminomas and four seminoma-matched controls, six non-seminomas and the corresponding matched controls, one non-GCT testis, one lymphoma, one endometrial carcinoma and one placenta sample.

**Fig. 5:**
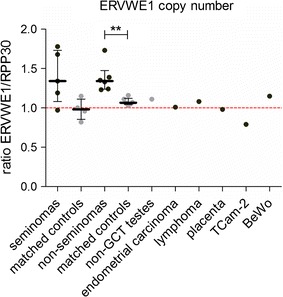
ERVWE1 copy number analysis. The ERVWE1 copy number was measured by ddPCR in the selected *panel* of human tumors. The copies of ERVWE1 were measured relatively to copies of the reference gene RPP30. Each sample is represented by a *dot*. The red spotted line schematically represents one copy per haploid genome. Each sample was measured in technical duplicate. Significance was assigned as follows: ** for P-values <0.01

The analyzed tumor-matched controls, the sample of non-GCT testis, and the sample of placenta did not show any abnormalities in the ERVWE1 copy number. On the other hand, seminomas displayed ERVWE1 copy numbers varying between 0.97 and 1.78 per haploid genome (Fig. 5). Despite higher levels of full-length ERVWE1 and spliced syncytin-1 mRNA expression in seminomas over the non-seminomas (Fig. 2), the ERVWE1 copy number in both types of GCTs did not differ (median 1.34 copies per haploid genome in both cases, Fig. 5). Furthermore, the ERVWE1 copy number in seminomas was not significantly higher in comparison with the seminoma-matched controls. On the other hand, the ERVWE1 copy number in the non-seminomas was significantly increased over the matched controls (Fig. 5). Moreover, the increased ERVWE1 copy number in individually analyzed GCTs mostly did not correspond to the ERVWE1 expression levels in these tumors, as documented e.g. for T3 and T36 tumor samples (Additional file 7: Fig. S5).

In conclusion, we detected increased ERVWE1 copy numbers in several GCTs. However, the higher ERVWE1 copy number did not correspond to the expression level of ERVWE1 RNA or syncytin-1 mRNA. Therefore, the increased ERVWE1 copy number was not sufficient to explain ERVWE1 derepression in GCTs.

### GCM1 is not overexpressed in GCTs or seminomas

**Fig. 6:**
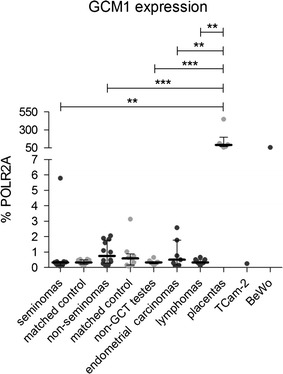
Human GCM1 mRNA expression analysis. The human GCM1 mRNA level was measured by qRT-PCR. All the data were normalized to % POLR2A expression. Each sample is represented by a *dot* and was measured as a technical triplicate. In each column, median with interquartile range is depicted. Significance was assigned as follows: *** for P-values <0.001, ** for P-values <0.01

Transcription factor GCM1 is known to stimulate ERVWE1 transcription. We explored the absolute levels of GCM1 in the GCTs using qRT-PCR. In the panel of randomly selected seminomas and non-seminoma GCTs, we detected similarly low levels of GCM1 expression (median 0.3350% POLR2A and 0.7493% POLR2A, respectively, Fig. 6) that was not increased over the tumor-matched controls and non-GCT testes. As expected, high levels of GCM1 mRNA were found only in the placentas (median 91% POLR2A) and BeWo cell line (57% POLR2A, Fig. 6). We concluded that high levels of ERVWE1 expression in seminomas were not mediated by the GCM1 transcription factor.

### Low level of CpG methylation and hydroxymethylation of ERVWE1 promoter in seminoma

We have shown previously that DNA hypermethylation suppresses transcription from the ERVWE1 promoter in non-placental tissues [12, 13] and we assume that demethylation is a prerequisite for ERVWE1 derepression. Recently, we have described elevated expression of TET enzymes in GCTs, particularly seminomas [30]. We therefore explored the levels of 5-mC and 5-hmC at the ERVWE1 promoter using bisulfite sequencing and oxidative bisulfite modification. The combination of these techniques allows discrimination between 5-mC and 5-hmC.

**Fig. 7:**
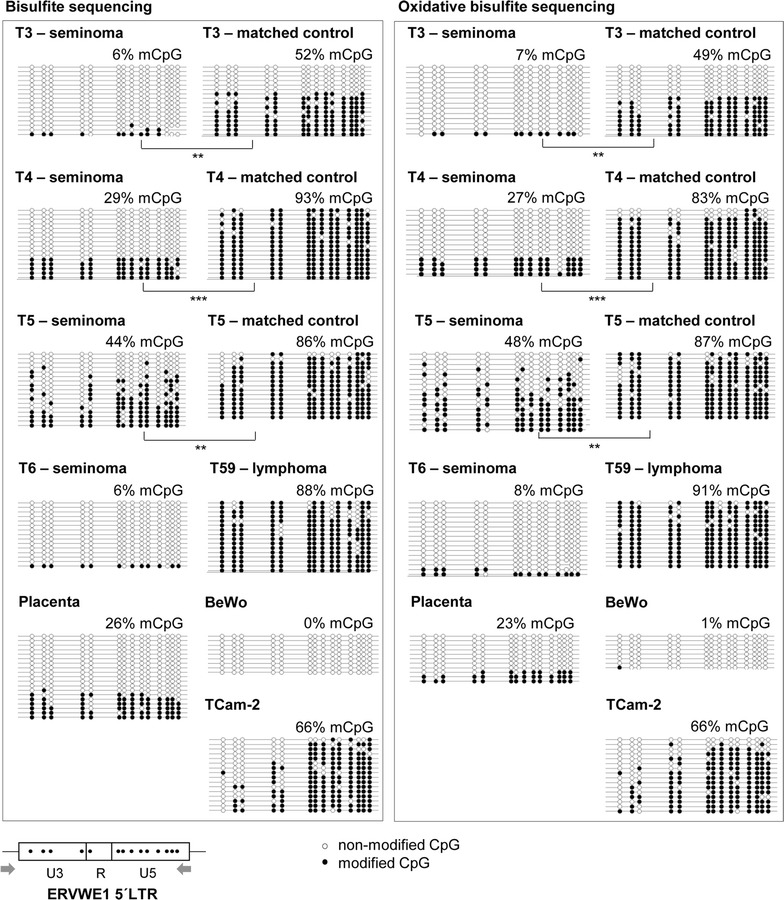
CpG methylation and hydroxymethylation profiles of the ERVWE1 promoter in selected samples. To analyze CpG modifications of the ERVWE1 promoter (5′LTR), bisulfite together with oxidative bisulfite sequencing were applied. Results of the bisulfite sequencing (5-mC + 5-hmC) are shown in the left part of the picture, results of the oxidative bisulfite sequencing (5-mC) in the right part of the picture. Analysis of the promoter molecules is shown as a linear array of open circles representing non-modified CpG residues and closed circles representing methylated or hydroxymethylated CpG residues. Each line represents one sequenced molecule of the 5′ LTR. The mean percentage of modified CpGs is presented for each sample. Schematic representation of the CpG dinucleotide distribution in the ERVWE1 promoter together with primers used for the analysis is shown at the bottom. Significance was assigned as follows: *** for P-values <0.001, ** for P-values <0.01

In accordance with our assumption, we found significantly decreased levels of 5-mCpG at the ERVWE1 promoter in all analyzed seminoma samples in comparison with the seminoma-matched controls (Fig. 7). The ERVWE1 promoter in seminoma T6, where the matched control was not available, was extremely hypomethylated (6% of mCpG, Fig. 7). No significant difference was observed between the results of bisulfite and oxidative bisulfite sequencing despite the efficient oxidative treatment (Additional file 8: Fig. S6). This indicates that very low or undetectable levels of 5-hmCpG were present at the ERVWE1 promoter in all seminoma samples at the time of analysis (Fig. 7; Table 1). The methylation patterns of seminomas T3, T4 and T6 showed either fully methylated or demethylated molecules (Additional file 9: Fig. S7). The analyzed lymphoma sample displayed hypermethylation of the ERVWE1 promoter and undetectable levels of 5-hmCpG (Fig. 7; Table 1). As expected, placenta and BeWo cell line contained a hypomethylated promoter of ERVWE1. Again, the 5-hmCpG was not detected (Fig. 7; Table 1).

**Table 1:** Percentage of 5-mCpG and 5-hmCpG on the ERVWE1 promoter

ERVWE1 promoter	Total 5-mCpG + 5-hmCpG^a^ Bisulfite sequencing %	5-mCpG^b^ Oxidative bisulfite sequencing %	5-hmC^c^ %
T3 (seminoma)	6	7	−1
T3-matched control	52	49	3
T4 (seminoma)	29	27	2
T4-matched control	93	83	10
T5 (seminoma)	44	48	−4
T5-matched control	86	87	−1
T6 (seminoma)	6	8	−2
T59 (lymphoma)	88	91	−3
Placenta	26	23	3
BeWo	0	1	−1
TCam-2	66	66	0

^a^The percentage of total 5-mCpG + 5-hmCpG modification of the ERVWE1 promoter was obtained after the conventional bisulfite sequencing

^b^The percentage of 5-mCpG modification of the ERVWE1 promoter was obtained after the oxidative modification of the bisulfite sequencing

^c^The percentage of 5-hmCpG modification of the ERVWE1 promoter was obtained by subtracting the percentage of 5-mCpG from the percentage of total modified CpG. Such subtraction produced negative values in several cases. The P value calculated using the Mann–Whitney two-tailed test was >0.05, i.e. non-significant in all samples

The results showed that 5-hmC levels at the ERVWE1 promoter in the GCTs were undetectable despite the high expression of TET1-3 mRNAs [30]. These findings pointed out the importance of 5-hmC turnover kinetics. Importantly, seminomas displayed low levels of 5-mC modification at the ERVWE1 promoter that allowed transcription from the ERVWE1 promoter to occur.

### Expression of additional endogenous retroviruses in GCTs

Finally, to assess whether derepression of endogenous retroviruses in seminomas is a more common phenomenon or whether it is ERVWE1-specific, we explored transcription of the HERV-H endogenous retroviral element co-localized with ERVWE1 on chromosome 7 (Fig. 1), in randomly selected samples of GCTs using ddPCR. We further quantified RNA levels of two additional non-pseudogenic HERV-W elements, localized on chromosomes 4 and 21 (Fig. 1), using the RT-qPCR approach. We selected these HERV-W loci because they were previously found overexpressed in testicular tumors using RNA microarray analysis [14].

**Fig. 8:**
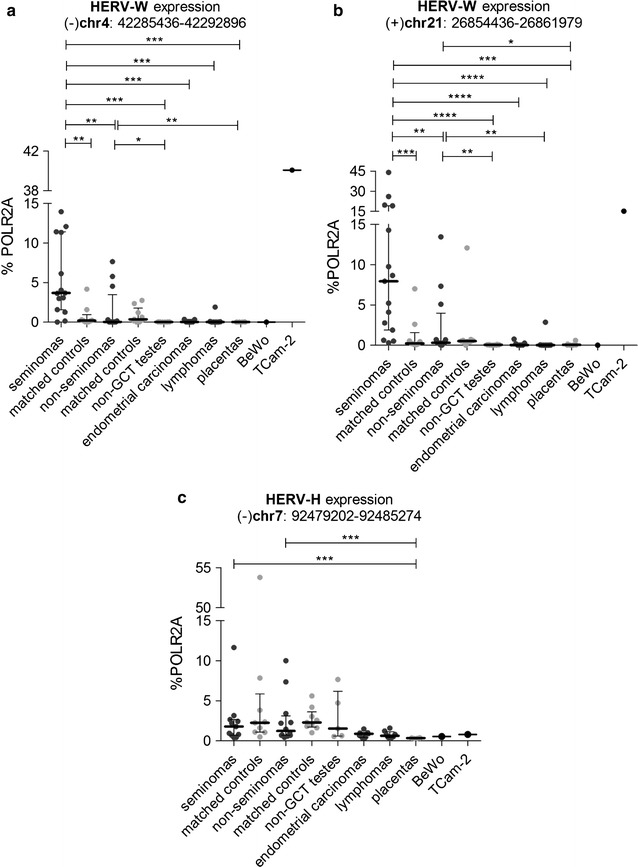
Expression of additional HERV-Ws and HERV-H in the panel of selected samples. Expression of HERV-W on chromosome 4 (**a**), HERV-W on chromosome 21 (**b**), and HERV-H adjacent to ERVWE1 (**c**), was analyzed in the panel of selected samples. All the data were normalized to % POLR2A. Each sample is represented by a dot and was measured as a technical triplicate. In each column, median with interquartile range is depicted. Significance was assigned as follows: **** for P-values <0.0001, *** for P-values <0.001, ** for P-values <0.01, * for P-values <0.05

Both analyzed endogenous HERV-W elements showed elevated expression in seminomas (median 4 and 8% of POLR2A for HERV-W on chromosomes 4 and 21, respectively) over the seminoma-matched controls and the non-seminoma GCTs (Fig. 8a, b). RNA transcribed from the HERV-W elements on chromosomes 4 and 21 further showed higher levels in seminomas in comparison with non-GCT testes, endometrial carcinomas, and lymphomas. Non-seminomas displayed higher levels of HERV-W on chromosomes 4 and 21 only over the non-GCT testes. The levels of HERV-H RNA were not significantly different in the sets of seminomas (median 1.8% of POLR2A), non-seminoma GCTs (1.2% of POLR2A), the respective tumor-matched controls (2.3% POLR2A), and non-GCT testes (1.5% of POLR2A, Fig. 8c). Interestingly, placenta samples efficiently silenced transcription of both HERV-W and HERV-H element (Fig. 8).

In conclusion, the expression of HERV-H located upstream to ERVWE1 and two additional HERV-W loci do not reach by far the levels of either full-length or spliced ERVWE1 RNA in seminomas (compare Figs. 2a, b and 8, see also the Additional file 6: Fig. S4). These results indicate that ERVWE1 is regulated independently of the other analyzed endogenous elements located either in close proximity to ERVWE1 or in remote genomic regions.

## Discussion

In this study, we have described elevated syncytin-1 mRNA expression in seminomas and mixed GCTs with a seminoma component. Our previous studies showed that ERVWE1 is suppressed by epigenetic mechanisms in non-placental tissues [12, 13]. This is further corroborated in this study showing the importance of ERVWE1 5′ LTR hypomethylation for its expression. Another mechanism indispensable for this aberrant upregulation in the seminoma was efficient splicing of ERVWE1 RNA providing the template for syncytin-1 translation. In summary, we show that the full-length and particularly the spliced form of ERVWE1 RNA is a hallmark of seminoma.

Although there are many examples of aberrant HERV expression in cancer (reviewed in [38]), ERVWE1 and ERVFRDE1 are exceptional in several aspects: expression of their envelope glycoproteins could have functional consequences for the cell and is, therefore, tightly controlled in non-placental tissues [11–14]. Second, only spliced transcripts can be used for envelope glycoprotein translation, which adds a further step to the complex control mechanisms [13]. Splicing efficiency has not been studied at all in cancer-specific aberrant expression of other HERV families. Third, the trophoblastic expression of ERVWE1 is controlled from a juxtaposed trophoblast-specific enhancer localized in MaLR LTR [9] and requires stimulation by certain tissue-specific transcription factors, such as GCM1 [10, 39], whose activity could be studied in germ line tumors. In neither type of tumors described in this study, we observed GCM1 expression comparable with placenta or BeWo cells, which points to the critical importance of promoter demethylation as a prerequisite for ERVWE1 transcriptional de-repression.

We did not observe elevated expression of ERVWE1 RNA in endometrial carcinomas as it was reported in [17]. Our collection of endometrial cancers was less extensive, we examined only seven endometrial carcinomas, including six endometrioid and one clear cell endometrial carcinomas of endometrial and myometrial localization, all in stage T1. Lymphomas showed slightly elevated levels of the full-length ERVWE1 RNA but only the background levels of the spliced syncytin-1 mRNA indicating very low splicing efficiency of the ERVWE1 RNA (Fig. 2). Comparison of the Hodgkin and non-Hodgkin lymphomas is shown in Additional file 4: Fig. S2.

The fusogenic capacity of Syncytin-1 and Syncytin-2 imposes the idea of syncytial activity in germ cell tumors with increased levels of spliced syncytin mRNA. We observed syncytial cells with low numbers of nuclei in the seminoma cell line TCam-2 as well as multinucleated syncytia in the BeWo cell line correlating well with the high expression of Syncytin-1 receptors, ASCT1 and ASTC2 in these cell lines. On the other hand, we did not observe systematic overexpression of ASCT2 in the GCTs. However, both seminomatous and, to a lesser extent, mixed GCTs are known to contain neoplastic giant cells of various types including mono- or multinuclear elements [40] with increased hCG expression [41]. It remains to be studied whether the presence of syncytia in GCTs requires syncytin-1 and -2 expression.

Interestingly, the methylation patterns of ERVWE1 promoter in several analyzed seminomas and placentas were bimodal showing either completely demethylated molecules or heavily methylated molecules. Only the T5 seminoma displayed a patchy methylation pattern of CpGs (Additional file 9: Fig. S7). This observation could underline the importance of promoter DNA demethylation for transcriptional derepression. A similar “all or nothing” pattern has also been observed in promoters of latent or activated HIV-1 proviruses [42]. Alternatively, different cellular types present in the samples could produce contrasting methylation patterns. The technical procedure of bisulfite sequencing and analysis used in this study was designed to avoid biased results by preferential amplification of single molecules and already adjusted for extremely rare targets [42].

In our recent work [30], we documented the hypomethylated genome of seminomas and associated this hypomethylation with strikingly elevated levels of TET dioxygenases, enzymes with DNA demethylase activities. Any decrease in DNA methylation affects particularly the repetitive part of the genome, as up to 90% of methylated CpGs are found in repetitive elements including the endogenous retroviruses [43]. Accordingly, there are reports on activation of HERVs and retrotransposons in 5-azacytidine-treated cells [44–46], embryonic stem cells [47], and induced pluripotent stem cells [48, 49]. ERVWE1, and to a lesser extent ERVFRDE1 derepression could be the direct consequence of the genome hypomethylation in seminoma cells. This, however, does not apply generally to all HERVs, and other proviral copies localized either in close proximity to ERVWE1 or on chromosomes 4 and 21 are not efficiently derepressed.

There are several reports of HERV-K expression in GCTs (e.g. [39]) and inducible expression of HERV-K *rec* gene in mice led to a disturbance of germ cell development reminiscent of early seminoma [40]. Genome-wide comparison of HERV expression in testicular cancer and normal testicular tissue has been done using copy-specific microarray and several HERV copies of families E, H, K, and W were found to be differentially expressed in testicular tumors. The expression of six HERV-W copies including ERVWE1 was confirmed by quantitative RT-PCR and the prevalence of spliced ERVWE1 transcripts was demonstrated. In comparison to non-testicular tumors and control healthy tissues, normal testicular tissue was not exceptional and expressed several ubiquitously represented HERV-E, H, and K copies.

Our finding of TET1 overexpression in seminomas [30] implicates the role of 5-hmC in transcriptional derepression. We, however, observed a very low or undetectable level of 5-hmC at the ERVWE1 promoter in seminomas examined in this study. Although unexpected, this paucity of 5-hmC is not at odds with previous reports. E.g., 5-hmC was detectable in early human PGCs but get gradually depleted later on [29]. The genic regions in the human PGCs showed a low but significant levels of 5-hmC whereas the retroelements showed very low levels of 5-hmC [28]. Because seminoma maintains multiple features of PGCs, this differential 5-hmC distribution could explain also the lack of 5-hmC on the ERVWE1 promoter.

The ERVFRDE1 expression was always lower than that of ERVWE1 resembling the low ERVFRDE1 level in placenta and BeWo cells (this study and [11]). Also, the level of DNA methylation at ERVFRDE1 5′LTR in healthy matched controls was rather low in comparison with ERVWE1 [13, 14]. On the other hand, splicing of ERVFRDE1 RNA was quite efficient despite low expression levels. Further research of the ERVFRDE1 promoter epigenetic status in the context of its transcriptional activation would improve our understanding of Syncytin-1 and Syncytin-2 contribution to cell-to-cell fusion.

## Conclusions

In summary, we analyzed ERVWE1 and ERVFRDE1 expression and splicing in a wide and well characterized set of germ cell tumors. Particularly, we correlated the ERVWE1 expression, low CpG methylation and, for the first time, also low CpG hydroxymethylation status of the ERVWE1 promoter with the whole genome hypomethylation phenotype of seminoma. We propose the spliced syncytin-1 expression as a marker of seminoma or seminomatous component of non-seminoma GCTs. As seminoma and non-seminoma treatments differ diametrically, we assume that detection of the spliced syncytin-1 mRNA could be useful for differential diagnosis and individualized therapy of GCTs. The elevated ERVWE1 expression without significantly increased copy number or increased level of GCM underscores the importance of epigenetic control of HERV-Ws. Identification of other HERV loci transcriptionally active in different types of GCTs would improve our understanding of endogenous retrovirus regulation.

## Additional files

**Additional file 1: Table S1.** List and characteristics of human cancer biopsies.

**Additional file 2: Table S2.** Primers used in the study.

**Additional file 3: Figure S1.** Expression analysis of ERVWE1 and ERVFRDE1 loci normalized to the TBP gene. Expression from the ERVWE1 (A, B) and ERVFRDE1 (C, D) loci was analyzed by qRT-PCR in the panel of tumor samples. Both the full-length RNA (A, C) and spliced mRNA (B, D) forms were quantified. All the data were normalized to the expression of TBP. Each sample is represented by a dot and was measured as a technical triplicate. In each column, median with interquartile range is depicted. Significance was assigned as follows: **** for P-values <0.0001, *** for P-values <0.001, ** for P-values <0.01, * for P-values <0.05.

**Additional file 4: Figure S2.** Expression analysis in Hodgkin and non-Hodgkin lymphomas. Expression of the full-length ERVWE1 RNA and spliced syncytin-1 mRNA, full-length ERVFRDE1 RNA and spliced syncytin-2 mRNA, ASCT1 mRNA, ASCT2 mRNA, GCM1 mRNA, HERV-H (chromosome 7) RNA, HERV-W (chromosome 4) RNA, HERV-W (chromosome 21) shown separately for Hodgkin and non-Hodgkin lymphomas. Significance was assigned as follows: * for P-values <0.05.

**Additional file 5: Figure S3.** Splicing efficiency of ERVWE1 and ERVFRDE1. Efficiency of splicing of the full-length ERVWE1 and ERVFRDE1 RNAs into the syncytin-1 and synctytin-2 mRNAs in seminomas and non-seminomas is depicted. The data were taken from the RT-qPCR analysis of the ERVWE1 and ERVFRDE1 expression (Figs. 2, 3). Each dot represents the efficiency of splicing in the analyzed sample. Median with interquartile range is depicted. Significance was assigned as follows: **** for P-values < 0.0001, *** for P-values <0.001.

**Additional file 6: Figure S4.** Comparison of the expression from ERVWE1, ERVFRDE1, HERV-W chromosome 4, HERV-W chromosome 21, and HERV-H chromosome 7 in seminomas and non-seminomas. Data from RT-qPCR analysis (Figs. 2, 3, and 8) were pooled for comparison of the expression from examined endogenous retroviral loci in seminomas (A) and non-seminomas (B). Each sample is represented by a dot and was measured as a technical triplicate. In each column, median with interquartile range is depicted. Significance was assigned as follows: **** for P-values <0.0001, ** for P-values <0.01, * for P-values <0.05.

**Additional file 7: Figure S5.** ERVWE1 copy number analysis in individual analyzed GCTs (A), and full-length ERVWE1 RNA and spliced syncytin-1 mRNA expression in individual GCTs (B). (A) The ERVWE1 copy number was measured by ddPCR in the selected panel of GCTs. T2 to T6 tumors are seminomas, T34 to T36, T41, and T42 GCTs are non-seminomas. The copies of ERVWE1 were measured relatively to the copies of the reference gene RPP30. Each sample is represented by a dot, black dots represent tumor samples, grey dots respective tumor-matched controls. The red spotted line schematically represents one copy per haploid genome. Each sample was measured in technical duplicate. (B) The expression from the ERVWE1 locus in the same panel of samples is depicted. For each sample, the levels of both full-length ERVWE1 RNA (white columns) and spliced syncytin-1 mRNA (black columns) are shown. The data are presented as the mean  ±  SD of technical triplicates. All the data were taken from the expression analysis of the ERVWE1 locus (Fig. 2).

**Additional file 8: Figure S6.** Control of oxidation efficiency at 5-hmC. The same region of the ERVWE1 promoter that was analyzed for 5-mC and 5-hmC modifications was amplified by PCR from genomic DNA employing the modified dNTP mix that contained dhmCTP instead of dCTP. The amplified product of 538 bp containing 155 5-hmCs was isolated from the agarose gel and 100 ng of the 5-hmC-containing fragment was subjected to oxidative bisulfite treatment. Thereafter, PCR was performed using the bisulfite-specific ERVWE1 primers (see Additional file 2: Table S2, primers ERVWE1-BIS-FW and RV). Amplification products of three primary PCRs were cloned in the pGEM-T-Easy vector and sequenced. Analysis was performed using the Quma software and all PCR clones were taken into account regardless of the C conversion rate. Overall 5-hmC to U conversion levels of 97.9–99.3% were observed in individual molecules of the oxidation controls indicating effective oxidation. Only the Cs in the CpG context are depicted: efficient oxidation of the 5-hmC to the 5-fC and subsequent bisulfite conversion is represented as an open circle while non-efficient oxidation plus bisulfite conversion of the 5-hmC is represented graphically as black circle. Two separately performed replicates are shown.

**Additional file 9: Figure S7.** Distribution of 5′ LTR molecules according to the proportion of methylated CpGs. Seminomas T3, T4, T5, T6, respective matched controls (when available), placenta sample, TCam-2 cell line, and lymphoma T59 are depicted. The total counts of methylated CpGs per molecule are shown on the Y-axis. The percentage of the respective molecules is shown on the X-axis.

## Abbreviations

ERVWE1: endogenous retrovirus WE1
ERVFRDE1: endogenous retrovirus FRDE1
ASCT1: alanine/serine/cysteine/threonine transporter 1
ASCT2: alanine/serine/cysteine/threonine transporter 2
GCM1: glial cells missing homolog 1
TET1: ten-eleven translocation methylcytosine dioxygenase 1
TET2: ten-eleven translocation methylcytosine dioxygenase 2
TET3: ten-eleven translocation methylcytosine dioxygenase 3
5-hmC: 5-hydroxymethylcytosine
5-mC: 5-methylcytosine
5-fC: 5-formylcytosine
GCT: germ cell tumor
PGC: primordial germ cell

## References

[1] Blaise S, de Parseval N, Benit L, Heidmann T (2003). Genomewide screening for fusogenic human endogenous retrovirus envelopes identifies syncytin 2, a gene conserved on primate evolution. Proc Natl Acad Sci USA.

[2] Frendo JL, Olivier D, Cheynet V, Blond JL, Bouton O, Vidaud M (2003). Direct involvement of HERV-W Env glycoprotein in human trophoblast cell fusion and differentiation. Mol Cell Biol.

[3] Mi S, Lee X, Li X, Veldman GM, Finnerty H, Racie L (2000). Syncytin is a captive retroviral envelope protein involved in human placental morphogenesis. Nature.

[4] Moller AMJ, Delaisse J-M, Soe K (2016). Osteoclast fusion: time-lapse reveals involvement of CD47 and syncytin-1 at different stages of nuclearity. J Cell Physiol..

[5] Lavillette D, Marin M, Ruggieri A, Mallet F, Cosset FL, Kabat D (2002). The envelope glycoprotein of human endogenous retrovirus type W uses a divergent family of amino acid transporters/cell surface receptors. J Virol.

[6] Marin M, Lavillette D, Kelly SM, Kabat D (2003). N-linked glycosylation and sequence changes in a critical negative control region of the ASCT1 and ASCT2 neutral amino acid transporters determine their retroviral receptor functions. J Virol.

[7] Grandi N, Cadeddu M, Blomberg J, Tramontano E (2016). Contribution of type W human endogenous retroviruses to the human genome: characterization of HERV-W proviral insertions and processed pseudogenes. Retrovirology.

[8] Pavlicek A, Paces J, Elleder D, Hejnar J (2002). Processed pseudogenes of human endogenous retroviruses generated by LINEs: their integration, stability, and distribution. Genome Res.

[9] Prudhomme S, Oriol G, Mallet F (2004). A retroviral promoter and a cellular enhancer define a bipartite element which controls env ERVWE1 placental expression. J Virol.

[10] Yu C, Shen K, Lin M, Chen P, Lin C, Chang GD (2002). GCMa regulates the syncytin-mediated trophoblastic fusion. J Biol Chem.

[11] Gimenez J, Montgiraud C, Oriol G, Pichon JP, Ruel K, Tsatsaris V (2009). Comparative methylation of ERVWE1/syncytin-1 and other human endogenous retrovirus LTRs in placenta tissues. DNA Res.

[12] Matouskova M, Blazkova J, Pajer P, Pavlicek A, Hejnar J (2006). CpG methylation suppresses transcriptional activity of human syncytin-1 in non-placental tissues. Exp Cell Res.

[13] Trejbalova K, Blazkova J, Matouskova M, Kucerova D, Pecnova L, Vernerova Z (2011). Epigenetic regulation of transcription and splicing of syncytins, fusogenic glycoproteins of retroviral origin. Nucleic Acids Res.

[14] Gimenez J, Montgiraud C, Pichon JP, Bonnaud B, Arsac M, Ruel K (2010). Custom human endogenous retroviruses dedicated microarray identifies self-induced HERV-W family elements reactivated in testicular cancer upon methylation control. Nucleic Acids Res.

[15] Vargas A, Moreau J, Landry S, LeBellego F, Toufaily C, Rassart E (2009). Syncytin-2 plays an important role in the fusion of human trophoblast cells. J Mol Biol.

[16] Liang CY, Wang LJ, Chen CP, Chen LF, Chen YH, Chen HW (2010). GCM1 regulation of the expression of syncytin 2 and its cognate receptor MFSD2A in human placenta. Biol Reprod.

[17] Strissel PL, Ruebner M, Thiel F, Wachter D, Ekici AB, Wolf F (2012). Reactivation of codogenic endogenous retroviral (ERV) envelope genes in human endometrial carcinoma and prestages: emergence of new molecular targets. Oncotarget.

[18] Strick R, Ackermann S, Langbein M, Swiatek J, Schubert SW, Hashemolhosseini S (2007). Proliferation and cell-cell fusion of endometrial carcinoma are induced by the human endogenous retroviral syncytin-1 and regulated by TGF-beta. J Mol Med.

[19] Moch H, Cubilla AL, Humphrey PA, Reuter VE, Ulbright TM (2016). The 2016 WHO classification of tumours of the urinary system and male genital organs-part A: renal, penile, and testicular tumours. Eur Urol.

[20] Almstrup K, Nielsen JE, Mlynarska O, Jansen MT, Jorgensen A, Skakkebaek NE (2010). Carcinoma in situ testis displays permissive chromatin modifications similar to immature foetal germ cells. Br J Cancer.

[21] Kristensen DG, Nielsen JE, Jorgensen A, Skakkebaek NE, Rajpert-De Meyts E, Almstrup K (2014). Evidence that active demethylation mechanisms maintain the genome of carcinoma in situ cells hypomethylated in the adult testis. Br J Cancer.

[22] Looijenga LH, Gillis AJ, van Gurp RJ, Verkerk AJ, Oosterhuis JW (1997). X inactivation in human testicular tumors. XIST expression and androgen receptor methylation status. Am J Pathol.

[23] Netto GJ, Nakai Y, Nakayama M, Jadallah S, Toubaji A, Nonomura N (2008). Global DNA hypomethylation in intratubular germ cell neoplasia and seminoma, but not in nonseminomatous male germ cell tumors. Mod Pathol.

[24] Peltomaki P (1991). DNA methylation changes in human testicular cancer. Biochim Biophys Acta.

[25] Smiraglia DJ, Szymanska J, Kraggerud SM, Lothe RA, Peltomaki P, Plass C (2002). Distinct epigenetic phenotypes in seminomatous and nonseminomatous testicular germ cell tumors. Oncogene.

[26] Wermann H, Stoop H, Gillis AJ, Honecker F, van Gurp RJ, Ammerpohl O (2010). Global DNA methylation in fetal human germ cells and germ cell tumours: association with differentiation and cisplatin resistance. J Pathol.

[27] Gkountela S, Zhang KX, Shafiq TA, Liao WW, Hargan-Calvopina J, Chen PY (2015). DNA demethylation dynamics in the human prenatal germline. Cell.

[28] Guo F, Yan L, Guo H, Li L, Hu B, Zhao Y (2015). The transcriptome and DNA methylome landscapes of human primordial germ cells. Cell.

[29] Tang WW, Dietmann S, Irie N, Leitch HG, Floros VI, Bradshaw CR (2015). A unique gene regulatory network resets the human germline epigenome for development. Cell.

[30] Benesova M, Trejbalova K, Kucerova D, Vernerova Z, Hron T, Szabo A (2017). Overexpression of TET dioxygenases in seminomas associates with low levels of DNA methylation and hydroxymethylation. Mol Carcinog..

[31] Ito S, D’Alessio AC, Taranova OV, Hong K, Sowers LC, Zhang Y (2010). Role of tet proteins in 5mC to 5hmC conversion, ES-cell self-renewal and inner cell mass specification. Nature.

[32] He YF, Li BZ, Li Z, Liu P, Wang Y, Tang Q (2011). Tet-mediated formation of 5-carboxylcytosine and its excision by TDG in mammalian DNA. Science.

[33] Ito S, Shen L, Dai Q, Wu SC, Collins LB, Swenberg JA (2011). Tet proteins can convert 5-methylcytosine to 5-formylcytosine and 5-carboxylcytosine. Science.

[34] Yamaguchi S, Shen L, Liu Y, Sendler D, Zhang Y (2013). Role of Tet1 in erasure of genomic imprinting. Nature.

[35] Kagiwada S, Kurimoto K, Hirota T, Yamaji M, Saitou M (2013). Replication-coupled passive DNA demethylation for the erasure of genome imprints in mice. EMBO J.

[36] Rengstl B, Newrzela S, Heinrich T, Weiser C, Thalheimer FB, Schmid F (2013). Incomplete cytokinesis and re-fusion of small mononucleated Hodgkin cells lead to giant multinucleated Reed-Sternberg cells. Proc Natl Acad Sci USA.

[37] Strissel PL, Ellmann S, Loprich E, Thiel F, Fasching PA, Stiegler E (2008). Early aberrant insulin-like growth factor signaling in the progression to endometrial carcinoma is augmented by tamoxifen. Int J Cancer.

[38] Kassiotis G (2014). Endogenous retroviruses and the development of cancer. J Immunol.

[39] Muroi Y, Sakurai T, Hanashi A, Kubota K, Nagaoka K, Imakawa K (2009). CD9 regulates transcription factor GCM1 and ERVWE1 expression through the cAMP/protein kinase A signaling pathway. Reproduction.

[40] von Hochstetter AR, Sigg C, Saremaslani P, Hedinger C (1985). The significance of giant cells in human testicular seminomas. Virchows Arch A.

[41] Butcher DN, Gregory WM, Gunter PA, Masters JR, Parkinson MC (1985). The biological and clinical significance of HCG-containing cells in seminoma. Br J Cancer.

[42] Trejbalova K, Kovarova D, Blazkova J, Machala L, Jilich D, Weber J (2016). Development of 5 LTR DNA methylation of latent HIV-1 provirus in cell line models and in long-term-infected individuals. Clin Epigenetics..

[43] Bestor TH, Tycko B (1996). Creation of genomic methylation patterns. Nat Genet.

[44] Roulois D, Yau HL, Singhania R, Wang YD, Danesh A, Shen SY (2015). DNA-demethylating agents target colorectal cancer cells by inducing viral mimicry by endogenous transcripts. Cell.

[45] Chiappinelli KB, Strissel PL, Desrichard A, Li HL, Henke C, Akman B (2015). Inhibiting DNA methylation causes an interferon response in cancer via dsRNA including endogenous retroviruses. Cell.

[46] Stengel S, Fiebig U, Kurth R, Denner J (2010). Regulation of human endogenous retrovirus-K expression in melanomas by CpG methylation. Gene Chromosome Cancer.

[47] Santoni FA, Guerra J, Luban J (2012). HERV-H RNA is abundant in human embryonic stem cells and a precise marker for pluripotency. Retrovirology.

[48] Friedli M, Turelli P, Kapopoulou A, Rauwel B, Castro-Diaz N, Rowe HM (2014). Loss of transcriptional control over endogenous retroelements during reprogramming to pluripotency. Genome Res.

[49] Fuchs NV, Loewer S, Daley GQ, Izsvak Z, Lower J, Lower R (2013). Human endogenous retrovirus K (HML-2) RNA and protein expression is a marker for human embryonic and induced pluripotent stem cells. Retrovirology.

