# Supplementary figures and images for: DNA hypomethylation and aberrant expression of the human endogenous retrovirus ERVWE1/syncytin-1 in seminomas
Source: Retrovirology. 2017 Mar 17;14:20. doi: 10.1186/s12977-017-0342-9 (PMC5356313; doi:10.1186/s12977-017-0342-9)

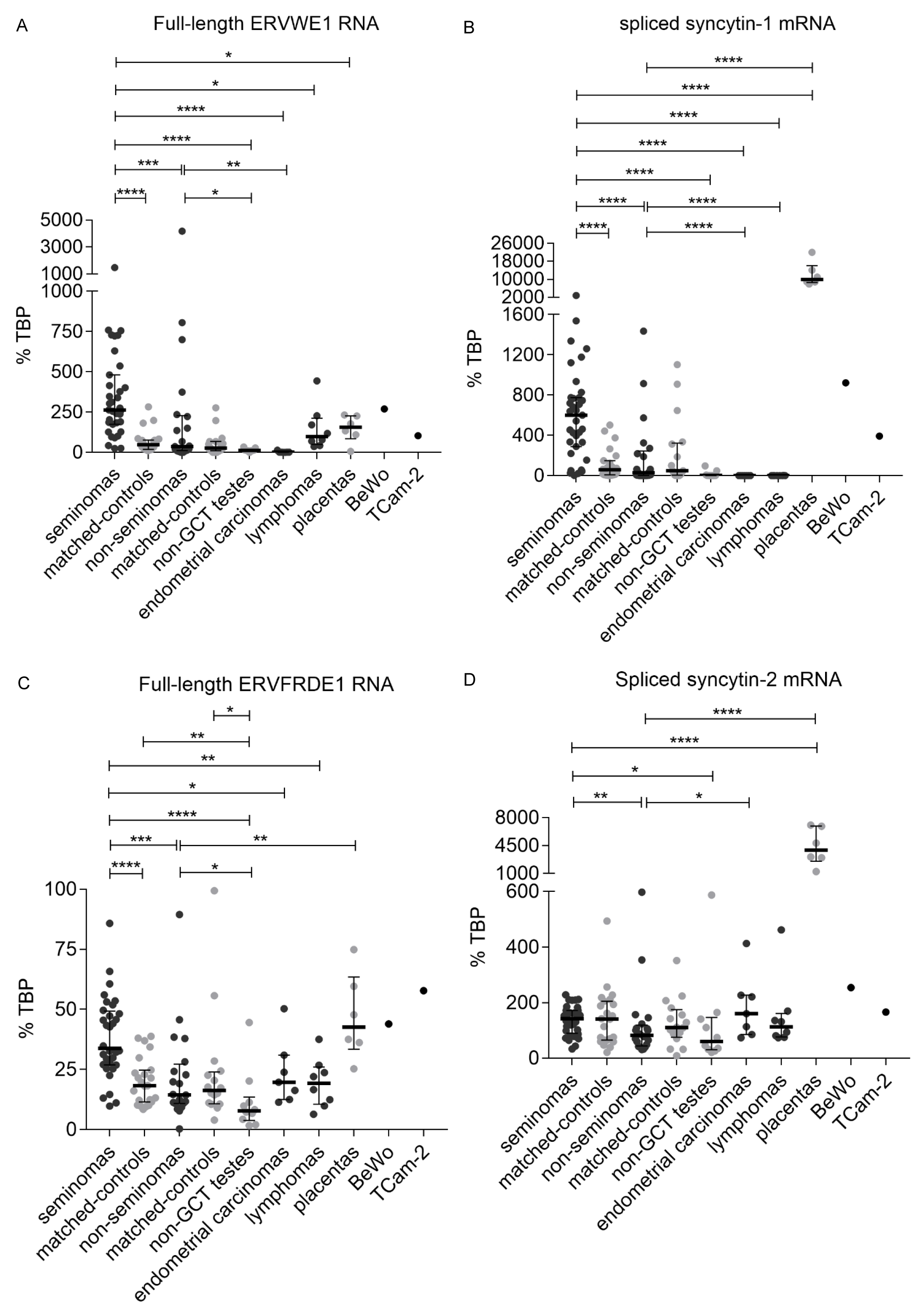

Supplement: Supplementary file 1 — Additional file 1: Table S1. List and characteristics of human cancer biopsies. [file 12977_2017_342_MOESM1_ESM.tif]

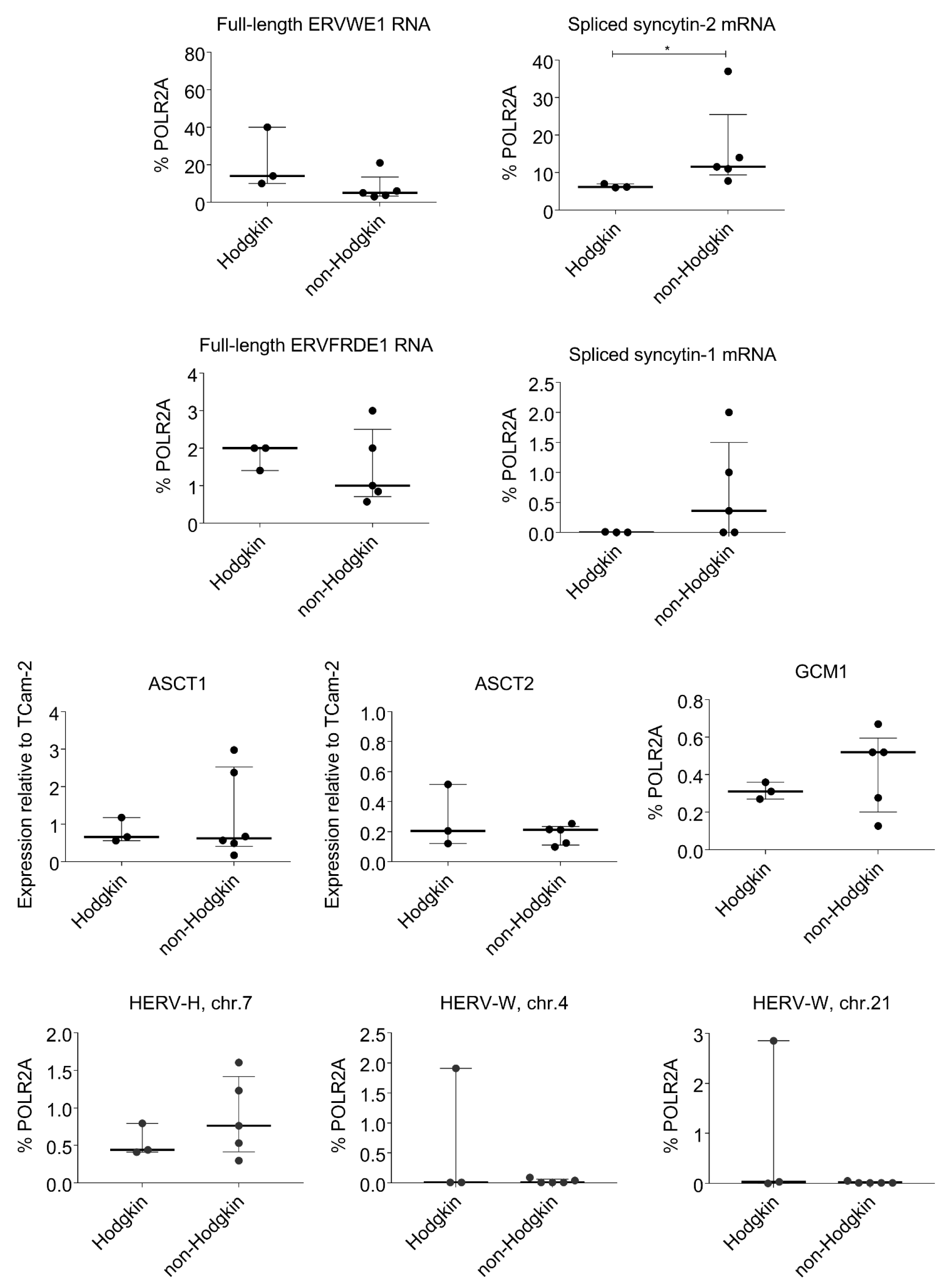

Supplement: Supplementary file 2 — Additional file 2: Table S2. Primers used in the study. [file 12977_2017_342_MOESM2_ESM.tif]

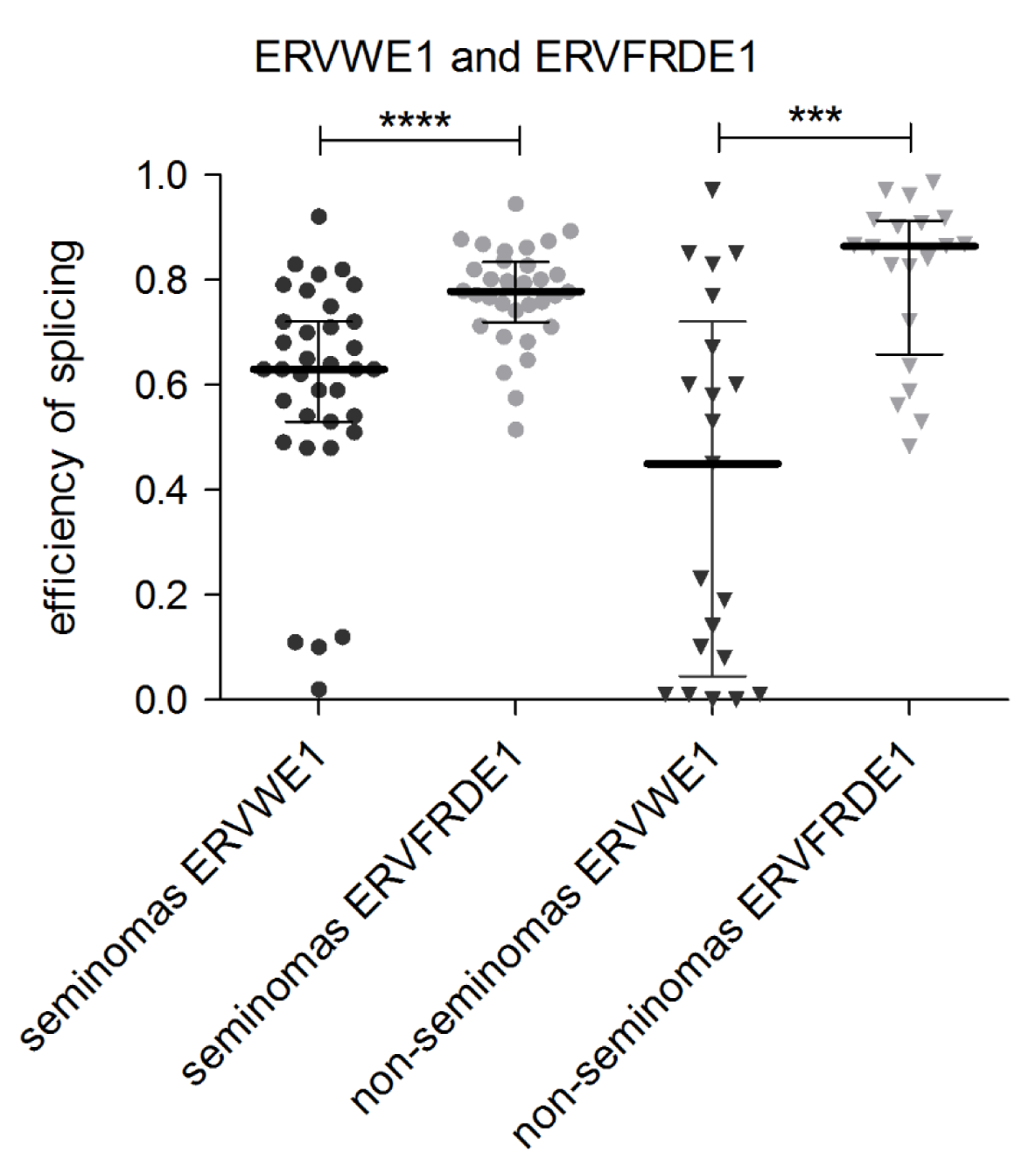

Supplement: Supplementary file 3 — Additional file 3: Figure S1. Expression analysis of ERVWE1 and ERVFRDE1 loci normalized to the TBP gene. Expression from the ERVWE1 (A, B) and ERVFRDE1 (C, D) loci was analyzed by qRT-PCR in the panel of tumor samples. Both the full-length RNA (A, C) and spliced mRNA (B, D) forms were quantified. All the data were normalized to the expression of TBP. Each sample is represented by a dot and was measured as a technical triplicate. In each column, median with interquartile range is depicted. Significance was assigned as follows: **** for P-values <0.0001, *** for P-values <0.001, ** for P-values <0.01, * for P-values <0.05. [file 12977_2017_342_MOESM3_ESM.tif]

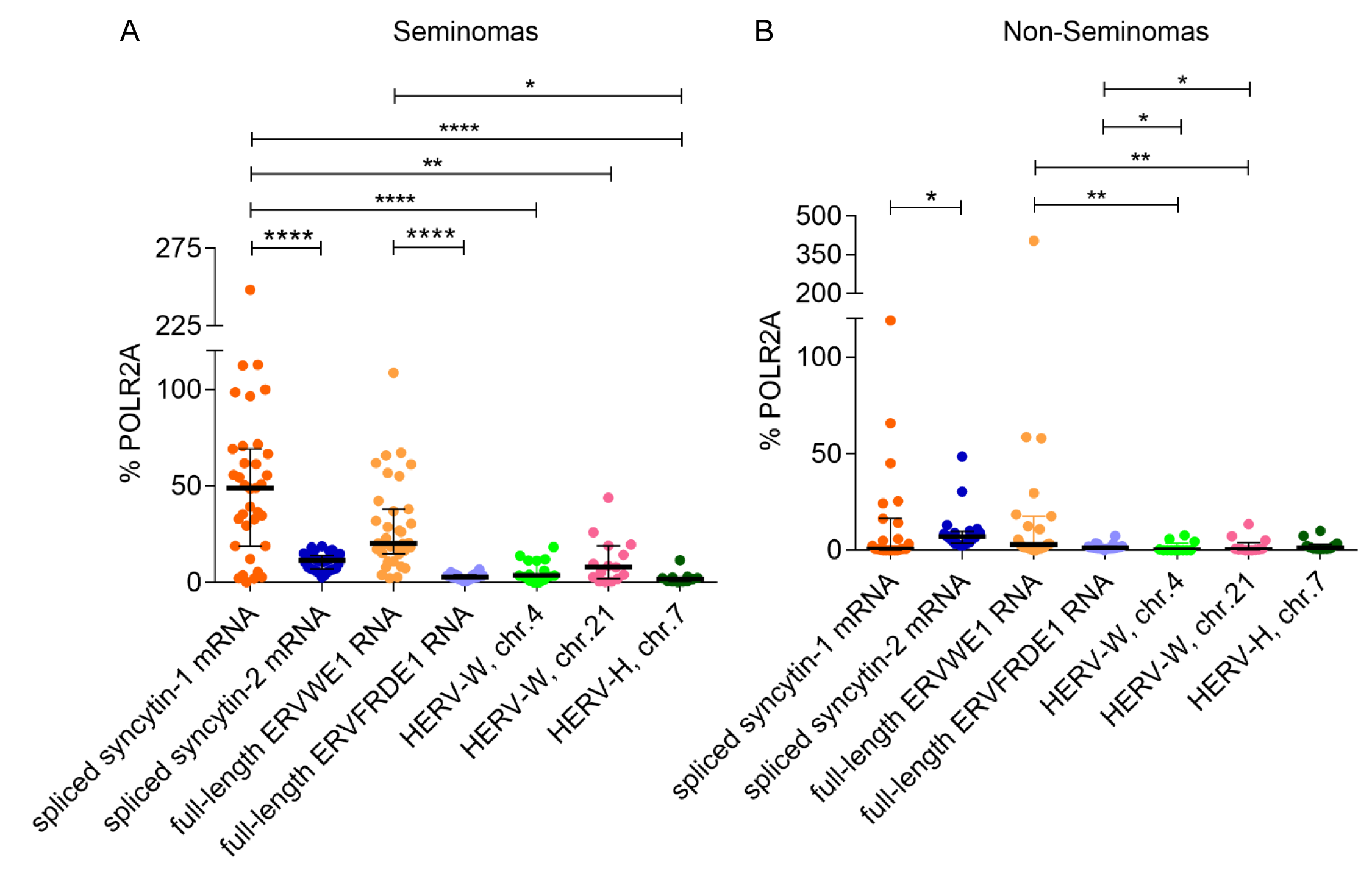

Supplement: Supplementary file 4 — Additional file 4: Figure S2. Expression analysis in Hodgkin and non-Hodgkin lymphomas. Expression of the full-length ERVWE1 RNA and spliced syncytin-1 mRNA, full-length ERVFRDE1 RNA and spliced syncytin-2 mRNA, ASCT1 mRNA, ASCT2 mRNA, GCM1 mRNA, HERV-H (chromosome 7) RNA, HERV-W (chromosome 4) RNA, HERV-W (chromosome 21) shown separately for Hodgkin and non-Hodgkin lymphomas. Significance was assigned as follows: * for P-values <0.05. [file 12977_2017_342_MOESM4_ESM.tif]

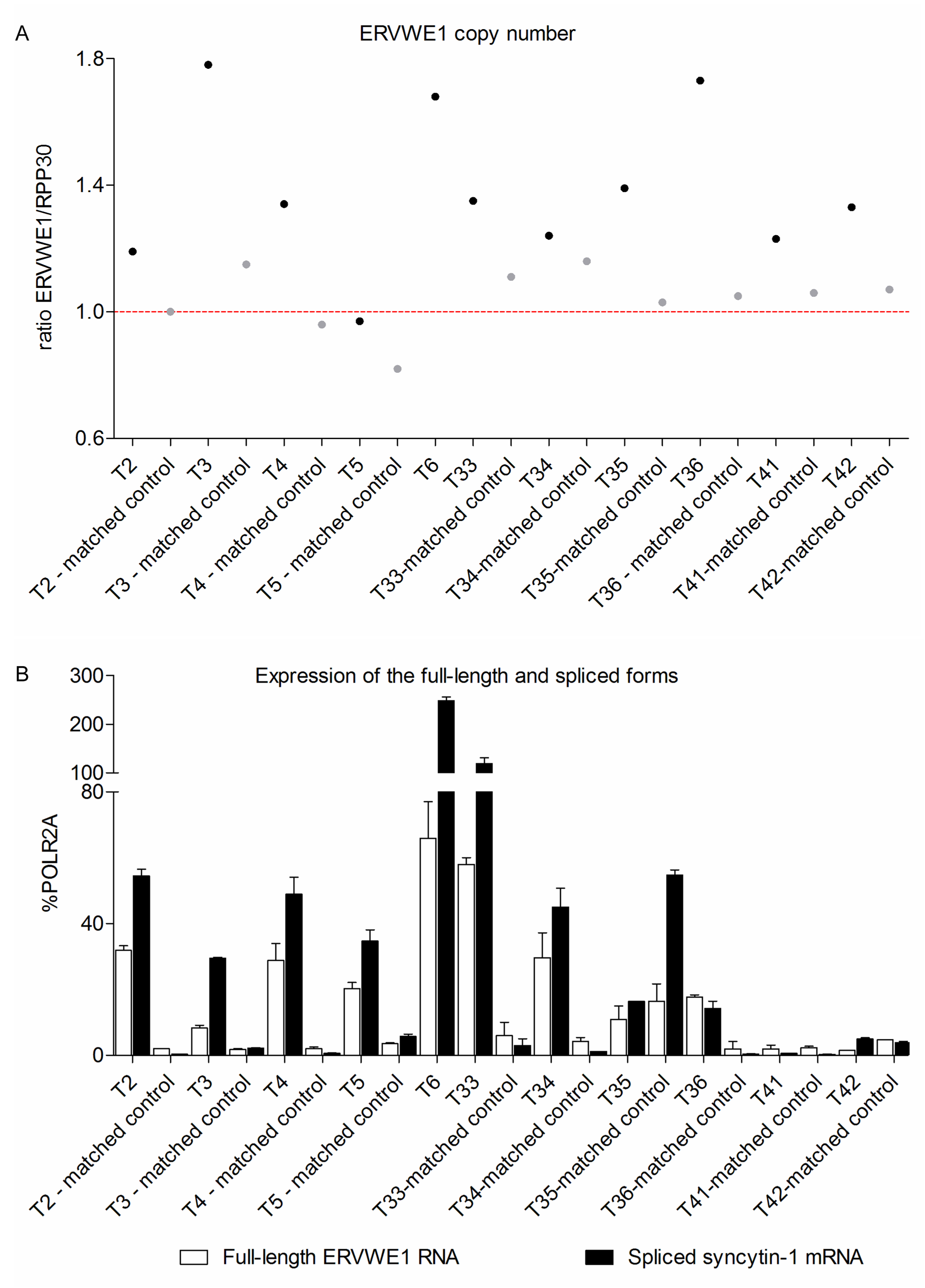

Supplement: Supplementary file 5 — Additional file 5: Figure S3. Splicing efficiency of ERVWE1 and ERVFRDE1. Efficiency of splicing of the full-length ERVWE1 and ERVFRDE1 RNAs into the syncytin-1 and synctytin-2 mRNAs in seminomas and non-seminomas is depicted. The data were taken from the RT-qPCR analysis of the ERVWE1 and ERVFRDE1 expression (Figs. 2, 3). Each dot represents the efficiency of splicing in the analyzed sample. Median with interquartile range is depicted. Significance was assigned as follows: **** for P-values < 0.0001, *** for P-values <0.001. [file 12977_2017_342_MOESM5_ESM.tif]

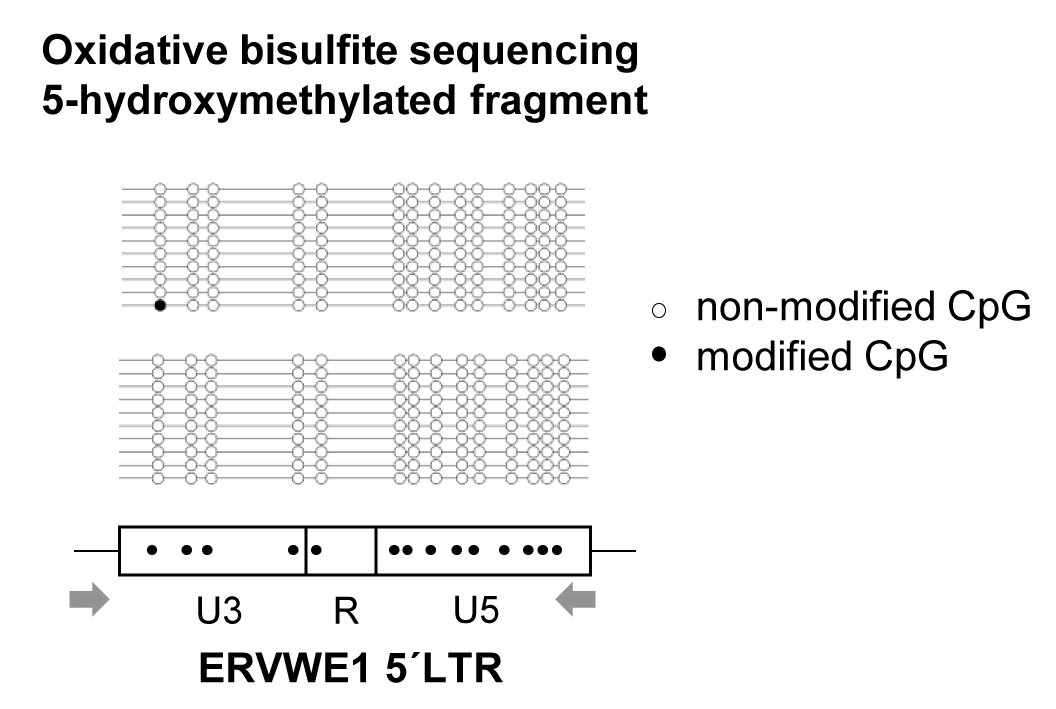

Supplement: Supplementary file 6 — Additional file 6: Figure S4. Comparison of the expression from ERVWE1, ERVFRDE1, HERV-W chromosome 4, HERV-W chromosome 21, and HERV-H chromosome 7 in seminomas and non-seminomas. Data from RT-qPCR analysis (Figs. 2, 3, and 8) were pooled for comparison of the expression from examined endogenous retroviral loci in seminomas (A) and non-seminomas (B). Each sample is represented by a dot and was measured as a technical triplicate. In each column, median with interquartile range is depicted. Significance was assigned as follows: **** for P-values <0.0001, ** for P-values <0.01, * for P-values <0.05. [file 12977_2017_342_MOESM6_ESM.tif]

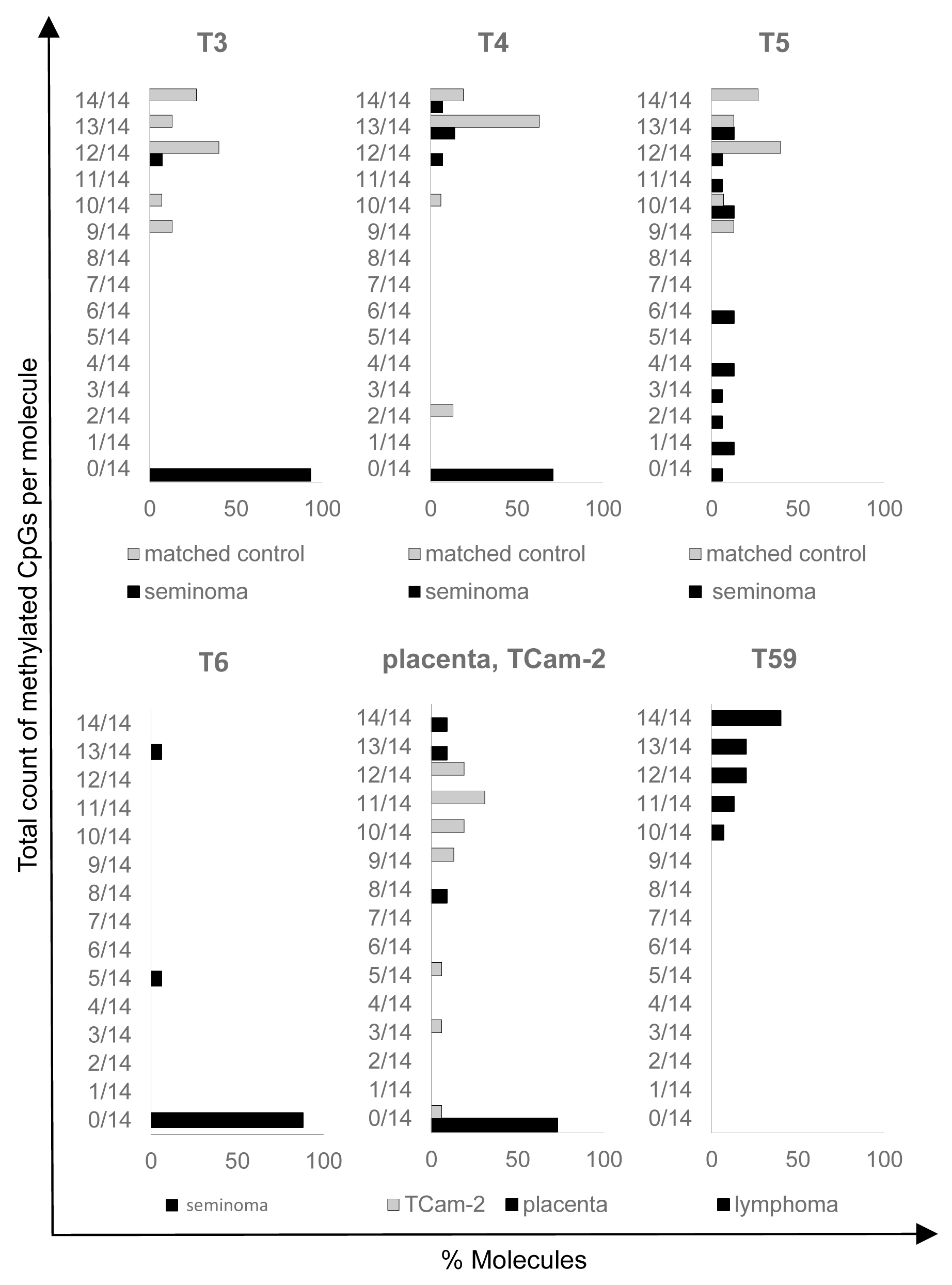

Supplement: Supplementary file 7 — Additional file 7: Figure S5. ERVWE1 copy number analysis in individual analyzed GCTs (A), and full-length ERVWE1 RNA and spliced syncytin-1 mRNA expression in individual GCTs (B). (A) The ERVWE1 copy number was measured by ddPCR in the selected panel of GCTs. T2 to T6 tumors are seminomas, T34 to T36, T41, and T42 GCTs are non-seminomas. The copies of ERVWE1 were measured relatively to the copies of the reference gene RPP30. Each sample is represented by a dot, black dots represent tumor samples, grey dots respective tumor-matched controls. The red spotted line schematically represents one copy per haploid genome. Each sample was measured in technical duplicate. (B) The expression from the ERVWE1 locus in the same panel of samples is depicted. For each sample, the levels of both full-length ERVWE1 RNA (white columns) and spliced syncytin-1 mRNA (black columns) are shown. The data are presented as the mean ± SD of technical triplicates. All the data were taken from the expression analysis of the ERVWE1 locus (Fig. 2). [file 12977_2017_342_MOESM7_ESM.tif]
