# Supplementary material for: DNA hypomethylation and aberrant expression of the human endogenous retrovirus ERVWE1/syncytin-1 in seminomas
Source: Retrovirology. 2017 Mar 17;14:20. doi: 10.1186/s12977-017-0342-9 (PMC5356313; doi:10.1186/s12977-017-0342-9)
Supplement: Supplementary file 8 — Additional file 8: Figure S6. Control of oxidation efficiency at 5-hmC. The same region of the ERVWE1 promoter that was analyzed for 5-mC and 5-hmC modifications was amplified by PCR from genomic DNA employing the modified dNTP mix that contained dhmCTP instead of dCTP. The amplified product of 538 bp containing 155 5-hmCs was isolated from the agarose gel and 100 ng of the 5-hmC-containing fragment was subjected to oxidative bisulfite treatment. Thereafter, PCR was performed using the bisulfite-specific ERVWE1 primers (see Additional file 2: Table S2, primers ERVWE1-BIS-FW and RV). Amplification products of three primary PCRs were cloned in the pGEM-T-Easy vector and sequenced. Analysis was performed using the Quma software and all PCR clones were taken into account regardless of the C conversion rate. Overall 5-hmC to U conversion levels of 97.9–99.3% were observed in individual molecules of the oxidation controls indicating effective oxidation. Only the Cs in the CpG context are depicted: efficient oxidation of the 5-hmC to the 5-fC and subsequent bisulfite conversion is represented as an open circle while non-efficient oxidation plus bisulfite conversion of the 5-hmC is represented graphically as black circle. Two separately performed replicates are shown. [file 12977_2017_342_MOESM8_ESM.docx]

**Additional File 1: Table S1. List and characteristics of human cancer biopsies.**

| **Tumor sample** | **Tumor- matched control** | **Type of tumor** | **Age** | **TNM** |
| --- | --- | --- | --- | --- |
| T1 | + | scar after SE^1^ | 51 | T1 N0 M0 S0 |
| T2 | + | pure SE | 31 | T1 N0 M0 S0 |
| T3 | + | pure SE | 36 | T2 N0 M0 S0 |
| T4 | + | pure SE | 49 | T2 N3 M0 S1 |
| T5 | + | pure SE | 52 | T1 N0 M0 S0 |
| T6 |  | pure SE | 36 | T1 N0 M0 S0 |
| T7 | + | pure SE | 42 | T1 N0 M0 S0 |
| T8 |  | pure SE | 36 | T2 N0 M0 S0 |
| T9 | + | pure SE | 67 | T1 N0 M0 S0 |
| T10 |  | pure SE | 50 | T2 N0 M0 S0 |
| T11 | + | pure SE | 32 | T1 N0 M0 S0 |
| T12 |  | pure SE | 40 | T1 N0 M0 S0 |
| T13 | + | pure SE | 32 | T1 N1 M0 S0 |
| T14 | + | pure SE | 30 | T1 Nx Mx S0 |
| T15 | + | pure SE | 42 | T2 N0 M0 S0 |
| T16 | + | pure SE | 44 | T1 Nx Mx Sx |
| T17 |  | pure SE | 29 | T1 N0 M0 S0 |
| T18 |  | pure SE | 34 | T2 N0 M0 S0 |
| T19 | + | pure SE (bilateral orchiectomy – left testis)^2^ | 31 | T2 Nx Mx Sx |
| T20 | + | pure SE (bilateral orchiectomy – right testis) | 31 | T2 Nx Mx Sx |
| T21 | + | pure SE | 28 | T1 N0 M0 S0 |
| T22, T22-B^3^ |  | pure SE | 46 | T1 Nx Mx Sx |
| T23 | + | pure SE | 50 | T1 N0 M0 S0 |
| T24 | + | pure SE | 26 | T1 Nx Mx Sx |
| T25 | + | pure SE | 28 | T2 N0 M0 S0 |
| T26 | + | pure SE | 29 | T1 Nx Mx Sx |
| T27 | + | pure SE | 43 | T1 Nx Mx Sx |
| T28 | + | pure SE | 43 | T1 N0 M0 S0 |
| T29, T29-B | + | pure SE | 39 | T3 N0 M0 S0 |
| T30, T30-B | + | pure SE | 53 | T1 N0 M0 S0 |
| T31, T31-B | + | pure SE | 45 | T1 N0 M0 S0 |
| T32 | + | mixed GCT – 80% SE, 20% EC^4^ | 51 | T1 N0 M0 S0 |
| T33 | + | mixed GCT – 60 – 70 % SE, 25 – 30% TE^5^, 15% EC | 43 | T1 N0 M0 S0 |
| T34 | + | mixed GCT – 80% SE, 20% EC | 41 | T1 N0 M0 S0 |
| T35 | + | mixed GCT – 95% SE, 5% EC | 34 | T1 N0 M0 S0 |
| T36 | + | mixed GCT – 90% EC, 10% SE | 28 | T1 N2 M1a S0 |
| T37 | + | mixed GCT – 40% EC, 25% SE , 25% YST^6^, 5% TE | 45 | T2 N0 M0 S1 |
| T38, T38-B | + | mixed GCT – 50% EC, 20% TE, 20% SE, 5% CHC^7^ | 26 | T1 N0 M0 S0 |
| T39, T39-B | + | mixed GCT – 60% TE, 30% SE, 10% EC, 1% YST | 56 | T2 N0 M0 S0 |
| T40 | + | mixed GCT – 85% YST, 15% SE | 36 | T1 N0 M0 S0 |
| T41 | + | mixed GCT – 40% YST, 35% EC, 25% TE | 36 | T1 N1 M0 S1 |
| T42, T42-B | + | mixed GCT – 60% CHC, 30% EC, 10% YST | 26 | T3 N0 M0 S0 |
| T43, T43-B | + | mixed GCT – 40% CHC, 30% EC, 30% YST | 28 | T1 N0 M0 S1 |
| T44 | + | pure EC | 37 | T1 Nx Mx Sx |
| T45 | + | pure EC | 24 | T2 N0 M0 S0 |
| T46 | + | pure TE with somatic type malignancy | 33 | T2 N0 M0 S0 |
| T47 |  | pure TE | 47 | T1 N0 M0 S0 |
| T48, T48-B | + | pure YST | 39 | T2 N0 M1b S1 |
| T49 |  | atrophic testis due to perinatal torsion | 32 |  |
| T50 |  | atrophic testes due to chronic purulent periorchitis | 80 |  |
| T51 |  | testicular necrosis due to testicular torsion | 56 |  |
| T52 |  | testes without GCT, testis with normal spermatogenesis | 44 |  |
| T53 |  | atrophic undescended testis | 52 |  |
| T54 |  | testicular ischemia-reperfusion injury | 45 |  |
| T55 |  | testes without GCT | 52 |  |
| T56 |  | atrophic testis due to testicular torsion | 78 |  |
| T57, T57-B |  | atrophic undescended testis | 21 |  |
| T58 |  | testicular ischemia-reperfusion injury | 45 |  |
| T59 |  | diffuse large B-cell non-Hodgkin lymphoma NOS^8^ | 61 | IIIE^‡^ |
| T60 |  | diffuse large B-cell non-Hodgkin lymphoma NOS | 71 | IV^‡^ |
| T61 |  | Hodgkin lymphoma, nodular sclerosis subtype | 26 | IIA^x^ |
| T62 |  | Hodgkin lymphoma, nodular sclerosis subtype | 78 | IVB^x^ |
| T63 |  | Hodgkin lymphoma, nodular sclerosis subtype | 61 | IIA^x^ |
| T64 |  | diffuse large B-cell non-Hodgkin lymphoma NOS | 59 | II2^‡^ |
| T65 |  | diffuse large B-cell non-Hodgkin lymphoma NOS | 80 | II2E^‡^ |
| T66 |  | follicular B-cell non-Hodgkin lymphoma grade IIIb, transformation to diffuse large B-cell lymphoma | 60 | IV^‡^ |
| T67 |  | endometrioid endometrial carcinoma, G3^9^ | 66 | T1a N0 M0 |
| T68 |  | endometrioid endometrial carcinoma, G3 | 65 | T1a N0 M0 |
| T69 |  | endometrioid endometrial carcinoma, G3 | 57 | T1b N0 M0 |
| T70 |  | endometrioid endometrial carcinoma, G3 |  |  |
| T71 |  | endometrioid endometrial carcinoma with small component of serous carcinoma, G3 | 77 | T1a Nx M0 |
| T72 |  | endometrioid endometrial carcinoma, G3 | 69 | T1b Nx M0 |
| T73 |  | clear cell endometrial carcinoma, G3 | 85 | T1a Nx M0 |
| placenta 1 |  | healthy terminal placenta |  |  |
| placenta 2 |  | healthy terminal placenta |  |  |
| placenta 3 |  | healthy terminal placenta |  |  |
| placenta 4 |  | healthy terminal placenta |  |  |
| placenta 5 |  | healthy terminal placenta |  |  |
| placenta 6 |  | healthy terminal placenta |  |  |

^1^SE seminoma; ^2^T19 and T20 samples were taken from the bilateral orchiectomy of the same patient; ^3^-B the second biopsy from different place of the same tumour ^4^EC embryonal carcinoma; ^5^TE teratoma; ^6^YST yolk sac tumour; ^7^CHC choriocarcinoma; ^8^NOS non-otherwise specified; ^9^G3 grade 3: poorly differentiated.

^‡^Classification according to modified Ann Arbor staging classification (Musshoff K. Clinical Staging Classification of Non-Hodgkin’s Lymphomas. Strahlentherapie; 1977. p. 218-221.)

^x^Classification according to Cotswold revision of the Ann Arbor staging classification of Hodgkin’s lymphomas (Lister TA, Crowther D, Sutcliffe SB, et al. Report of a Committee Convened to Discuss the Evaluation and Staging of Patients with Hodgkin’s Disease: Cotswolds meeting. J. Clin. Oncol. 1989; 7:1630-1636. Erratum in J. Clin. Oncol. 1990; 8:1602.)
