# Supplementary material for: DNA hypomethylation and aberrant expression of the human endogenous retrovirus ERVWE1/syncytin-1 in seminomas
Source: Retrovirology. 2017 Mar 17;14:20. doi: 10.1186/s12977-017-0342-9 (PMC5356313; doi:10.1186/s12977-017-0342-9)
Supplement: Supplementary file 9 — Additional file 9: Figure S7. Distribution of 5′ LTR molecules according to the proportion of methylated CpGs. Seminomas T3, T4, T5, T6, respective matched controls (when available), placenta sample, TCam-2 cell line, and lymphoma T59 are depicted. The total counts of methylated CpGs per molecule are shown on the Y-axis. The percentage of the respective molecules is shown on the X-axis. [file 12977_2017_342_MOESM9_ESM.docx]

**Additional File 2: Table S2. Primers used in the study.**

| **Primer indication** | **Sequence** | **Annealing Temperature** |
| --- | --- | --- |
| ERVWE1 FW | 5´ ACATTTTGGCAACCACGAAC 3´ | 55°C qRT-PCR  57°C ddPCR |
| Syncytin-1 spliced RV | 5´ GGCCATGGGGATTTATGATT 3´ | 55°C qRT-PCR |
| ERVWE1 non-spliced RV | 5´ AAAGTGGAAGCTGGCTTGAG 3´ | 55°C qRT-PCR  57°C ddPCR |
| ERVWE1 non-spliced PROBE | 5´ FAM-AGACTCAGGTGTGAGGCTATCTGGG-BHQ1 3´ | 57°C ddPCR |
| ERVFRDE1 FW | 5´ CAAGTCAAGGGCTGAACAGG | 55°C |
| Syncytin-2 spliced RV | 5´ CGGTAGGCTGCTAGTGAAGG 3´ | 55°C |
| ERVFRDE1 non-spliced RV | 5´ CAGAGCCACTGTGGTTGAGA 3´ | 55°C |
| GCM1 FW | 5´ GCCAGATTCCTATGCCAAAC 3’ | 58°C |
| GCM1 RV | 5’ CAAGGGATGAGCTTCAGAGG 3’ | 58°C |
| RPII FW | 5´ GCACCACGTCCAATGACAT 3´ | 55 – 59°C |
| RPII RV | 5´ GTGCGGCTGCTTCCATAA 3´ | 55 – 59°C |
| RPII PROBE | 5’ HEX-TACCACGTCATCTCCTTTGATGGCTCCTA-BHQ1 3’ | 55 – 59°C |
| ASCT1 FW | 5’ TTTGCGACAGCATTTGCTAC 3’ | 55°C |
| ASCT1 RV | 5’ CGCTGTGGCAGTCACTAGAA 3’ | 55°C |
| ASCT2 FW | 5’ CATCATCCTCGAAGCAGTCA 3’ | 55°C |
| ASCT2 RV | 5’ AGTGTTTGAGGAGGGGGTTT 3’ | 55°C |
| HERVH FW | 5´ CGTGGCTGCAGTACAAACTTGATAA 3´ | 57°C |
| HERVH RV | 5´ GGTCTGTAGCAAAGGAGGATTCAAAG 3´ | 57°C |
| HERVH PROBE | 5´ FAM-CTTTAAATGGCCAGAATATGGCACTTTCA-BHQ1 3´ | 57°C |
| HERV-W-4-FW | 5´ GAGCTTTGGTCTGCCTGGAA3´ | 60°C |
| HERV-W-4-RV | 5´ TGCGCCACGATCTCAACTGT 3´ | 60°C |
| HERV-W-21-FW | 5´ AGGCAGAAAGCTGTCGTCCG 3´ | 60°C |
| HERV-W-21-RV | 5’ GGGGCTACACTTTCAAGAAAGTCATC 3’ | 60°C |
| RPP30 FW | 5’ GATTTGGACCTGCGAGCG 3’ | 55-60°C |
| RPP30 RV | 5’ GCGGCTGTCTCCACAAGT 3’ | 55-60°C |
| RPP30 PROBE | 5’ HEX-TTCTGACCTGAAGGCTCTGCGC-BHQ1 3’ | 55-60°C |
| ERVWE1 copy No FW | 5’ CGCCTGGAGATACAGCAATTA 3’ | 60°C |
| ERVWE1 copy No RV | 5’ CAGCTAGGCTTAGGGATTCTTAG 3’ | 60°C |
| ERVWE1 copy No PROBE | 5’ FAM-TGAGAGACAGGACTAGCTGGATTTCCT-BHQ1 3’ | 60°C |
| ERVWE1-BIS-FW | 5’ GGAGATATAGTAATTATTTTGTAATTGAGAGAT 3’ | 54-58^o^C |
| ERVWE1-BIS-RV | 5’ AACAATAACAAACCTTTAACCCAATTA 3’ | 54-58^o^C |
| ERVWE1-nonBIS-FW | 5’ GGAGATACAGCAATTATCTTGCAACTGAGAGAC 3’ | 60°C |
| ERVWE1-nonBIS-RV | 5’ AACAATGGCAAGCCTTTAGCCCAATTG 3’ | 60°C |
| TBP FW | 5’ GAGCTGTGATGTGAAGTTTCC 3’ | 60°C |
| TBP RV | 5’ TCTGGGTTTGATCATTCTGTAG 3’ | 60°C |
